# 2D/3D Halide Perovskites for Optoelectronic Devices

**DOI:** 10.3389/fchem.2021.715157

**Published:** 2021-08-19

**Authors:** Xiang Chen, Hai Zhou, Hao Wang

**Affiliations:** Hubei Yangtze Memory Labs, School of Microelectronics and Faculty of Physics and Electronic Science, Hubei University, Wuhan, China

**Keywords:** 3D perovskite, 2D perovskite, solar cells, light emitting diodes, photodetectors

## Abstract

The traditional three-dimensional (3D) halide perovskites (HPs) have experienced rapid development due to their highly power conversion efficiency (PCE). However, the instability of 3D perovskite on humidity and UV irradiation blocks their commercialization. In the past few years, two-dimensional (2D) halide perovskites attract much attention because they behave better stability due to the water resistance of the aliphatic carbon chains in the 2D perovskite lattice. In this review, we categorize the 2D/3D perovskites based on the applications [i.e., solar cells (SCs), light-emitting diodes (LEDs) and photodetectors (PDs)]. We further discuss the recent efforts in the performance enhancement of the 2D/3D perovskite-based devices. However, there are still some difficulties before 2D/3D HPs is fully commercialized. We will provide some scientific and technical challenges and prospects in the article to point out the future direction.

## Introduction

Organic-inorganic HPs have attracted great attention for applications in SCs, PDs and LEDs, because of their excellent optoelectronic properties, such as long carrier diffusion length, high defect tolerance, high light trapping ability, high quantum yield, high mobility and low non-radiation recombination (Yan et al., 2018). In particular, organic-inorganic hybrid perovskites such as MAPbI_3_ have been widely used in SCs and their record power conversion efficiency (PCE) has reached above 25.5% in these few years (NREL, 2020).

Although these great progresses, the commercialization of the perovskite-based devices still faced significant challenges, one of which is that the perovskite is very sensitive to moisture. Recently, some work showed that reducing the dimension of perovskite from 3D to 2D structure is a promising strategy for enhancing resistivity to humidity due to the water repellent nature of the aliphatic carbon chains in the 2D perovskite lattice (Liang et al., 2008; Lin et al., 2019). But there are some disadvantages. Firstly, undesired orientations cause charge transport problems, such as more complex losses and bigger charge accumulation. Secondly, the band gap increases, leading to further deviations from the ideal band gap of a single junction solar cell. In recent years, some groups have made lots of efforts to overcome these disadvantages and improved the device performance. Liu and coauthors mixed the 2D and 3D halide perovskites and achieved the high-stability SCs with the PCE of up to 20.08% (Liu et al., 2019). The non-toxic solvent processing and device stability are indispensable developments in the industrialization of organometallic HPs SCs. At the same time, the preparation of large-scale with high-efficiency devices is also a challenge (Lin et al., 2019).

In this review, we emphasize the progress in 2D/3D hybrid HPs on SCs, as well as a brief introduction to their progress in LEDs and PDs. Firstly, we introduce the basic structure and characteristics of the 3D and 2D perovskite. Secondly, we introduce and discuss the latest progress in the three types of optoelectronic devices, and finally technical challenges and prospects are provided to guide the future direction.

## 2D/3D Perovskite Materials

### Structure of 2D/3D Perovskite Materials

**FIGURE 1:**
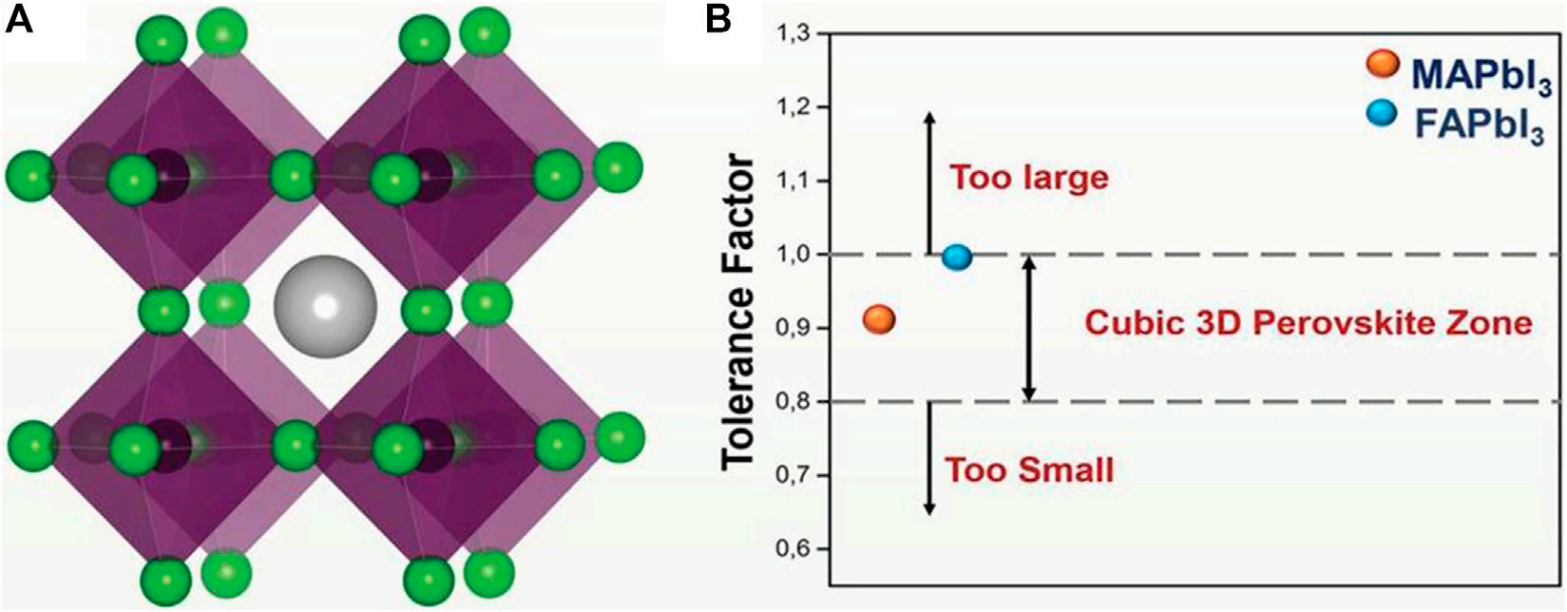
**(A)** Crystal structure of cubic perovskite with ABX_3_; **(B)** TF for formation of ideal cubic perovskite structure. Reproduced with permission from Krishna et al., 2018. Copyright 2018 Advanced Functional Materials.

Perovskite refers to a class of ceramic oxides whose molecular formula is ABO_3_. A site is a generally alkaline earth or rare earth element ion, and the B site is a transition element ion such as Ti, Mn, Co, Fe, etc. This kind of oxide was first discovered as a calcium titanate (CaTiO_3_) compound in perovskite ores, which belong to orthorhombic system (Galuskin et al., 2008; Ji et al., 1994). The 3D halide perovskite is characterized by the molecular formula ABX_3_, as shown in Figure 1A, A = organic or inorganic monovalent cation (such as MA^+^ (asmethylammonium), FA^+^(formazan) or Cs^+^) at the apex angle of the face-centered cubic lattice, B = divalent cation (Dou et al., 2015; Völker et al., 2015) (Pb^2+^ or Sn^2+^), located in the body of the cubic crystal, X = halogen (Cl^−^, Br^−^ or I^−^) in the face-centered (Han et al., 2018). Compared with organic semiconductor materials (organic dyes, conjugated polymers), organometallic HPs materials exhibit special electrical and optical characteristics because of their unique quantum confinement structure: 1) exciton binding energy is small. For example, the exciton binding energy of CH_3_NH_3_PbI_3_ is only 19 ± 3 meV (Sun et al., 2014). Therefore, most of the excitons generated by the excitation of light can be separated as free electrons and holes at room temperature (RT). For organic semiconductor materials, their exciton binding energy is generally higher than 250 mV, so a higher driving force is required to effectively separate the free careers. 2) The Bohr radius of the exciton is large. The Bohr radii of CH_3_NH_3_PbBr_3_ and CH_3_NH_3_PbI_3_ are 20 and 22 Å (Tanaka et al., 2003), respectively, but the organic semiconductor material is about 1 Å. 3) The dielectric constant is large. For CH_3_NH_3_PbBr_3_ and CH_3_NH_3_PbI_3_, the dielectric constants are 4.8 and 6.5, respectively. But the dielectric constant of organic semiconductor materials is mostly low (∼2–4), so the charge separation is difficult and the recombination phenomenon is severe, limiting the improvement of photoelectric performance. 4) The carrier has a high diffusion rate with a long diffusion distance. Owing to the small effective mass of electrons and holes generated in CH_3_NH_3_PbI_3_ (Stoumpos et al., 2013), the electron and hole mobility can reach 7.5 and 12.5–66 cm·V^−^^1^·s^−1^, respectively (Stoumpos et al., 2013). The diffusion length of electrons and holes varies with the crystal structure, up to the order of micrometers (Stranks et al., 2013), much higher than the diffusion distance of excitons in organic semiconductor materials (∼10 nm). 5) The absorption range is wide and the absorption coefficient is high. For CH_3_NH_3_PbI_3_, it is a direct bandgap semiconductor with an absorption edge of about 800 nm and a band gap of 1.5 eV (Baikie et al., 2013). The light absorption coefficient of CH_3_NH_3_PbI_3_ at 360 nm is as high as 4.3 × 10^5^ cm^−^^1^ (Sun et al., 2014), which is much higher than that of organic semiconductor materials [10 (Liang et al., 2008) cm^−1^]. These characteristics of perovskite materials enable them to fully absorb sunlight during operation and reduce energy loss during photoelectric conversion, making them excellent in the SCs with various structures. The remarkable characteristic of perovskite SCs is that they have high open circuit voltage (V_oc_) and short circuit current (J_sc_). The V_oc_ of the SCs based on CH_3_NH_3_PbI_3_ perovskite absorber layer is generally 0.8–1.0 V, and the maximum can exceed 1.1 V (Yella et al., 2011; Lee et al., 2012; Zhou et al., 2014). The V_oc_ can increase to 1.3 V by using CH_3_NH_3_PbBr_3_ with a larger band gap as the light absorbing layer (Edri et al., 2013). In addition, the high light absorption coefficient of perovskites and the long distance of exciton diffusion determine that the SCs can output a higher current, which can exceed 20 mA/cm^2^ (Xiao et al., 2014a; Wojciechowski et al., 2014; Zhou et al., 2014).

Goldschmidt’s Tolerance Factor (TF) is used to calculate the deformation of the perovskite crystal structure to predict the 3D to 2D transition (Krishna et al., 2018). TF depends only on the ionic radius of the A, B and X sites of the perovskite structure, where $\text{t }=\left({\text{R}}_{\text{A}}{\;+\text{ R}}_{\text{X}}\right)/\left[\sqrt{2}\left({\text{R}}_{\text{B}}{\;+\text{ R}}_{\text{X}}\right)\right]$. Under normal conditions, ① when 0.7 < *t* < 0.9, the crystal structure is distorted, and the orthorhombic, square, and rhombohedral crystal systems with low symmetry are easily formed. At this time, the A or X ion is large; ② when 0.9 < *t* < 1, a cubic lattice with good stability and symmetry is generally formed, which is an ideal perovskite structure; ③ when *t* > 1, A ions are larger, which makes the 3D structure of perovskite to be 2D structure. Most prototype 3D perovskites, such as MAPbI_3_ or FAPbI_3_, have a value of 0.8 < *t* < 1 (Amat et al., 2014; Jacobsson et al., 2015; Collings et al., 2016) (Figure 1B). When the crystal structure is distorted, it results in octahedrons that share edges and face, rather than the original angular shared structure of the classic ABX_3_ perovskite. However, what we need to understand is that the factors affecting the stability of the crystal are non-geometric factors such as chemical stability and bond cost (Fan et al., 2015). Perovskites have these advantages, providing a prerequisite for the fabrication of perovskite materials with different sizes, dimensions and band gaps (Han et al., 2018).

**FIGURE 2:**
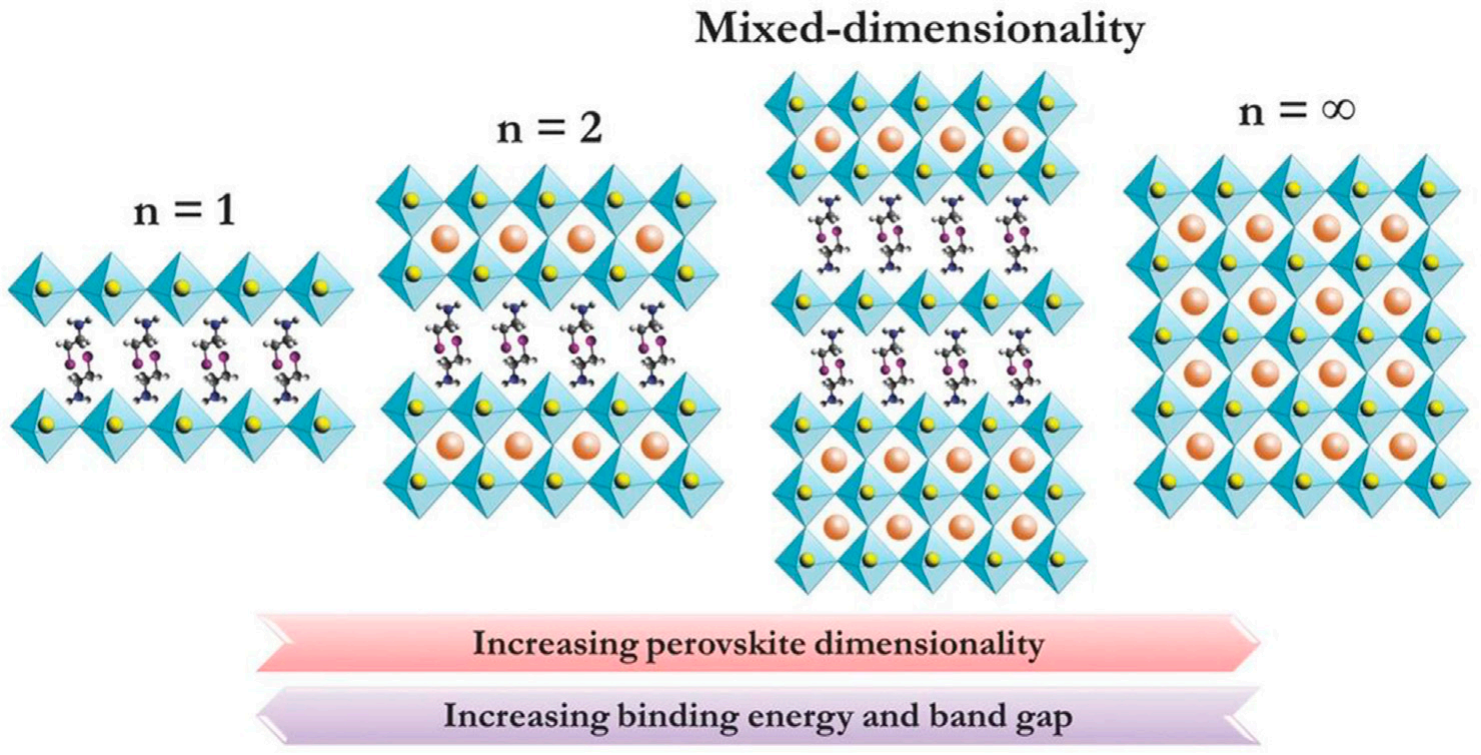
Figure 2 is a diagram showing the crystal structure of perovskites with different dimensions, such as 2D perovskite, mixed dimension 2D/3D perovskite and 3D perovskite. Reproduced with permission from Koh et al., 2016. Copyright 2016 Advanced Materials.

The 2D perovskite is formed by introducing an organic functional group into a 3D perovskite, using a Ruddlesden-Popper crystal structure with the formula (RNH_3_)_2_A_n-1_B_n_X_3n+1_, (*n* = 1, 2, 3, 4 ...), wherein (A_n−1_B_n_X_3n+1_)^2−^ represents a conductor layer derived from the parent 3D perovskite, such as cesium (Cs) lead iodide (CsPbI_3_). The conductor layer is isolated by R-NH_3_ [a large aliphatic or aromatic alkyl ammonium spacer cation such as butylammonium (BA) and phenethylammonium (PEA)]. *n* = ∞ constitutes a 3D structure; *n* = 1 corresponds to a pure 2D structure and when the value of n is others, it is a quasi-2D structure. The 2D structure is flexible due to the diversity of the *n* value and organic functional groups. Over the past few years, 2D perovskites have attracted much attention for the better stability than their 3D counterparts. However, by inserting an organic functional group into three dimensions, a 2D/3D hybrid structure can be formed, which has the advantages of two dimensions [Figure 2 shows 2D, 2D/3D HPs, 3D structural changes (Krishna et al., 2018)], showing a great attractive research direction for obtaining high-performance perovskite devices.

### Properties of 2D Perovskite Materials

The 2D perovskite material with a unique structure shows superior properties, which can be expressed in the following aspects.

Firstly, the 3D perovskite material MAPbI_3_ reacts with water and the reaction can be explained by four equations:

$$\mathrm{MAI+Pb}I_{2}↔\mathrm{MAPb}I_{3}$$ (1)

$$\mathrm{MAPb}I_{3}+H_{2}O↔\mathrm{MAPb}I_{3}\cdot H_{2}O$$ (2)

$$\mathrm{MAPb}I_{3}+H_{2}O↔\mathrm{MAPb}I_{3}\cdot H_{2}O$$ (3)

$$MA_{4}\mathrm{Pb}I_{6}\cdot 2H_{2}O↔\mathrm{Pb}I_{2}+4\mathrm{MAI+}2H_{2}O.$$ (4)

Compared with 3D perovskite, the organic spacer cation of 2D perovskite will increase the hydrophobicity of the perovskite structure. In addition, the spacer cations can slow down the second reaction, which makes the material less susceptible to moisture.

Secondly, the almost constant conductivity of the perovskite conductor layer and the conductive insulation of the organic spacer layer result in a natural multiple quantum well structure in which the organic spacer layer acts as a “wall” and the perovskite layer acts as a “well”. When a 2D material is excited, the binding energy is increased due to the quantum confinement effect, thereby forming excitons instead of electrons and holes. In addition, experiments have shown that the charge likes to travel along the surface of the iodide (“well transfer”). When the value of n becomes small, exciton absorption and peak emission peaks can be seen, and a blue shift phenomenon occurs. The presence of multiple quantum well structures is advantageous for the illumination of LEDs.

Moreover, in terms of structure, the 2D layered perovskite breaks through the strict limitation of the tolerance factor concept and exhibits good structural adjustability (Collings et al., 2016). Continuous tunability of exciton absorption/excitation energy according to quantum confinement effect can be achieved by changing n and R (Jin et al., 2014; Byun et al., 2016; Liu et al., 2017).

Finally, the most unique advantage of 2D compare to 3D is that the structure can be easily changed by changing the molecular design of the spacer cation. For example, changing the ammonium ion, the length of the alkyl chain, and the insertion of the π-conjugated fragment, etc. 2D perovskite has a wider range of applications due to its photoelectric properties and diversity of design composition. Moreover, the 2D material is well matched with the 3D lattice, which provides a possibility for 2D/3D mixing materials. In recent years, 2D/3D mixed halide perovskite has become more and more popular because it has the advantages of two dimensions, which provides the basis for the preparation of high PCE and high stability devices in the future.

## Progress of 2D/3D Mixed HPs in Optoelectronic Devices

The application and progress of 2D, quasi-2D and 2D/3D mixed HPs materials in SCs, LEDs and PDs are summarized below.

### Research Progress in SCs

The SC we know is also called photovoltaic device, which is a device that can convert light energy into electrical energy. The core parameters of SCs include PCE, J_sc_, V_oc_, FF (Fill factor), etc. (Koh et al., 2016; Han et al., 2018) In recent years, the PCE of the SCs by using 3D perovskite material has increased rapidly, but the stability was difficult to be solved. In contrast, the stability of 2D perovskite is much better than that of 3D perovskite. At the same time, 2D/3D HPs devices also show the characteristics of high stability and high PCE.

#### 2D HPs as a Light Absorbing Material

**FIGURE 3:**
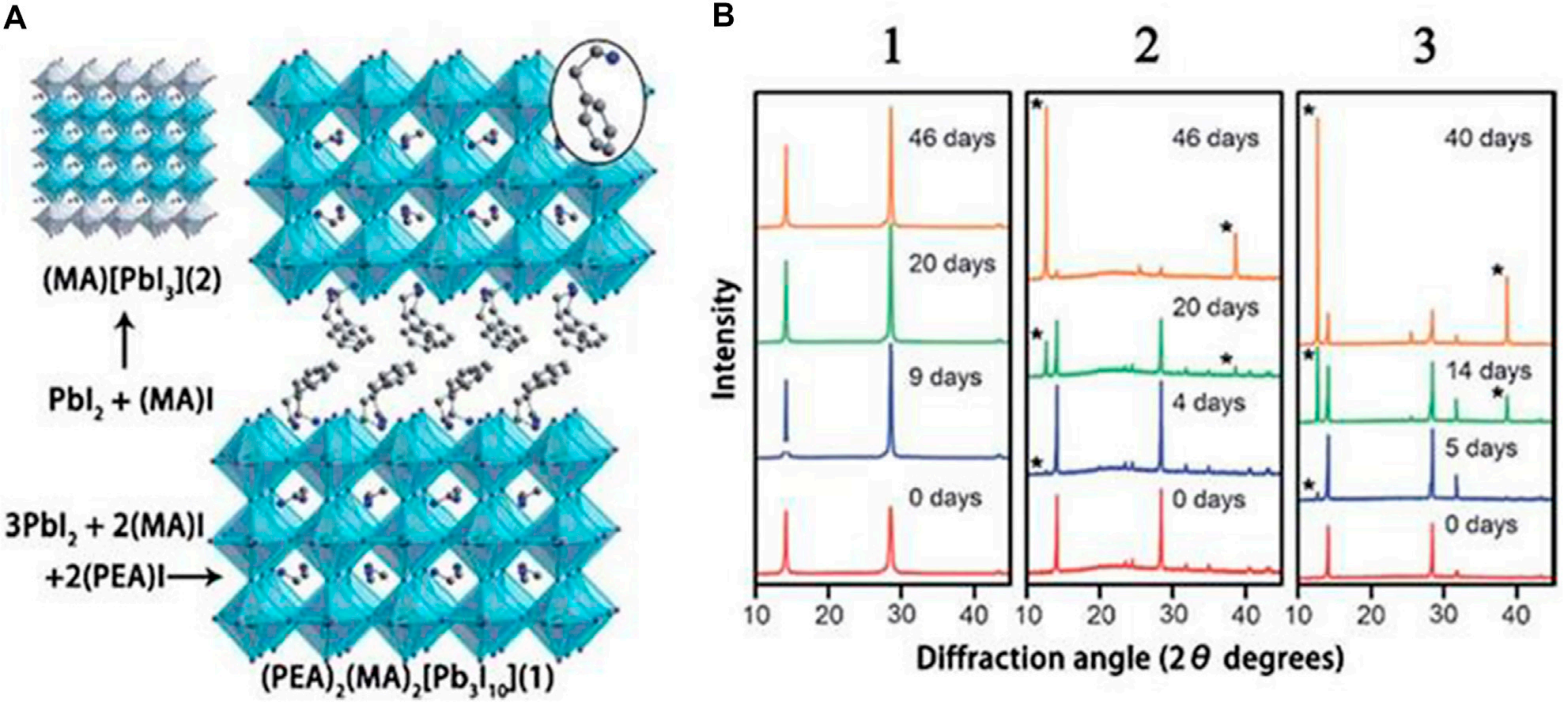
**(A)** Crystal structure of 3D HPs (MA)(PbI_3_) (2) and 2D HPs (PEA)_2_(MA)_2_(Pb_3_I_10_) (1); **(B)** XRD patterns of (PEA)_2_(MA)_2_(Pb_3_I_10_), (MA)(PbI_3_) and (MA)(PbCl_3_) powders after storage for different days in an air environment with a humidity of 52%. Reproduced with permission from Smith et al., 2014. Copyright 2014 Angewandte Chemie International Edition.

In 1992, Nurmikko and others first composited 2D perovskites ((PEA)_2_ (MA)_n−1_PbnI_3n+1)_) and studied their photoelectric performances. In 2014, Karunadasa and others showed the first application of this 2D HPs (Figure 3A) as a light absorber in planar SCs, yielding 4.73% PCE (Smith et al., 2014). Interestingly, this 2D HPs have excellent humidity tolerance, and the X-ray diffraction pattern remains substantially unchanged after storage for 46 days at a relative humidity of 52% (Figure 3B). Subsequently, Kanatzidis and others replaced the PEA with BA, and demonstrated a (BA)_2_(MA)_n−1_Pb_n_I_3n+1_ perovskite SC with a PCE of 4.02% (Cao et al., 2015). They found that when the *n* of the BA-based HPs increased from *n* = 1 to *n* = ∞, the band gap decreased from 2.24 to 1.52 eV and the band gap of the PEA based 2D HPs showed the same trend. However, they found that the BA-based 2D perovskites always showed a smaller band gap than the PEA-based counterparts, suggesting that the large spacer cations played a decisive role in the perovskite film.

**FIGURE 4:**
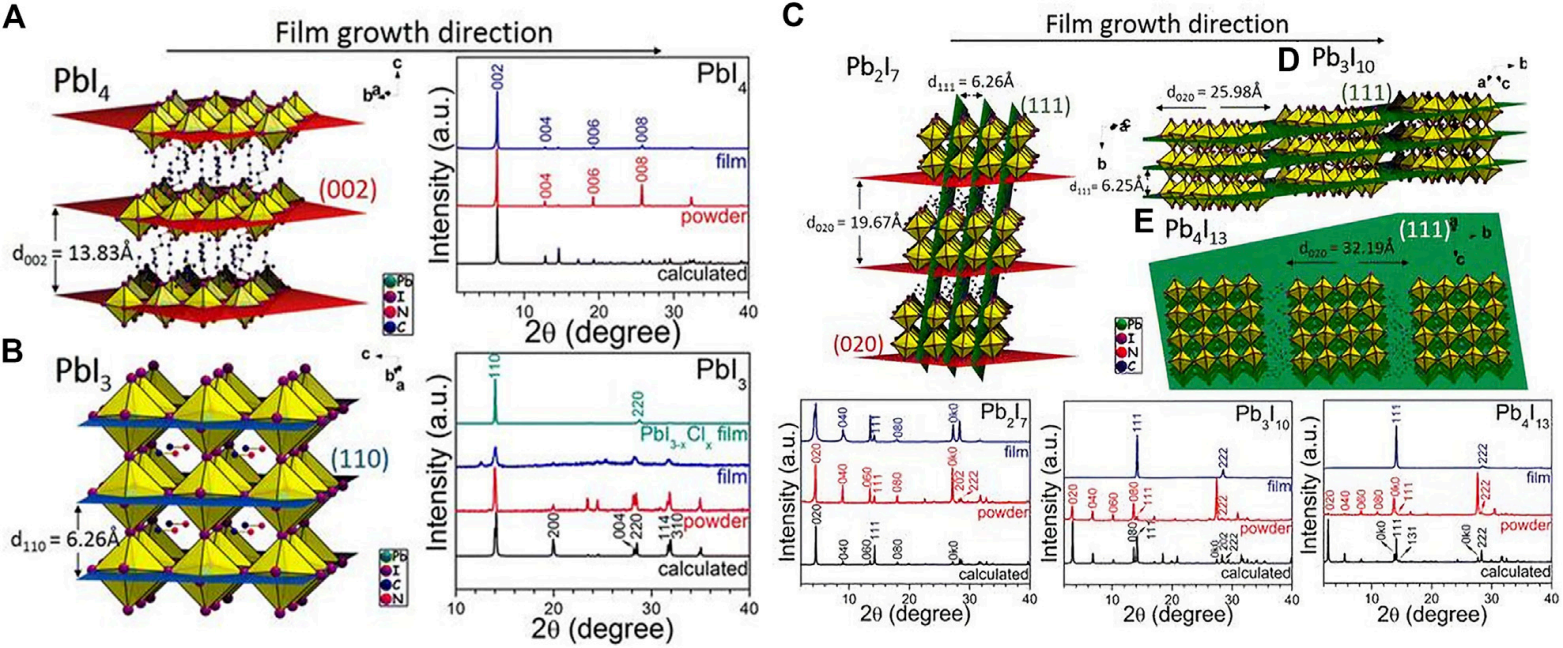
XRD patterns of **(A)** BA_2_PbI_4_, **(B)** MAPbI_3_, **(C)** (BA)_2_(MA)Pb_2_I_7_, **(D)** (BA)_2_(MA)_2_Pb_3_I_10_, and **(E)** (BA)_2_(MA)_3_Pb_4_I_13_ HPs and the illustration of their respective diffraction planes. Reproduced with permission from Cao et al., 2015. Copyright 2015 Journal of the American Chemical Society.

However, the PCE of the aforementioned 2D HPs materials is too low to be commercialized. Adjusting n and R to get different types of 2D and quasi-2D perovskite is a good way for high PCE based devices. When the value of n is large enough, the perovskite material can achieve effective charge extraction and sufficient light absorption, also the small n benefits to maintain the 2D nature. Kanatzidis and others reported that (BA)_2_(MA)_n−1_Pb_n_I_3n+1_ had a random orientation and an out-of-plane orientation change from in-plane orientation (Cao et al., 2015). When *n* = 1, the perovskite mainly grows along the (110) direction, revealing the growth in the horizontal direction [as shown in Figures 4A,B and the corresponding XRD pattern (whether it is a pure two-dimensional perovskite or a three-dimensional perovskite)]. Then when *n* > 1, the MA ions that grow and expand outside the layer will compete with the BA ions that grow in the plane layer. The XRD with *n* = 2 shows not only (0k0) reflections but also (111) and (202) reflections, which also reveals vertical growth (as shown in Figure 4C and the corresponding XRD). When *n* > 2, there will be no growth along the (0k0) plane (This (0k0) corresponds to (00k) in Figure 4A, because the initial coordinate axis direction of each figure is different) In addition, it is difficult to obtain the HPs of *n* > 4 with a pure phase, and thus a new preparation method is required.

Kanatzidis and others reported a near-single-crystal perovskite film by hot casting (HC) with a good out-of-plane preferential orientation (Tsai et al., 2016). This method effectively improves the charge transfer from the HPs film to the electrode, achieving the PCE from 4.02 to 12.52%. In addition to this high PCE, it showed slower degradation than 3D toward the light and relative humidity. Liu and others also used the hot casting method to obtain *n* = 4 quasi-2D HPs and PCE was close to 13.7% (Zhang et al., 2017a).

Knatzidis further improved the HC process by changing the proportion of dimethyl sulfoxide (DMSO) and N, N-dimethylformamide (DMF) to synthesize a higher n-value perovskite film (*n* = 5). It was found that the solvent without DMSO was evaporated extremely rapidly during the HC process, resulting in imperfect self-assembly of the HPs thick plate. If DMSO was added, an intermediate solvation phase could be formed, which slows down the crystallization rate. Finally, the optimized planar heterojunction SC device achieved 10% PCE (DMF: DMSO = 3:1) (Tsai et al., 2016).

The HC method makes it difficult to control the substrate temperature during the spin coating process, which is not conducive to large-area production. Liang’s team used short-branched iso-BA instead of BA as spacer cations to prepare (iso-BA)_2_(MA)_3_Pb_4_I_13_ HPs, which showed a significant increase in crystallinity even at room temperature (RT), and 8.82% of PCE was obtained. In addition, the unencapsulated film was able to maintain its original color after 840 h of storage at an ambient temperature of 20°C and an RH (Room humidity) of 60%, indicating its good stability (Chen et al., 2017). Zhou et al. changed the out-of-plane orientation growth by introducing 20% FA (formazan) to produce compound (BA)_2_(MA, FA)_3_Pb_4_I_13_ film, and PCE was as high as 12.81% (Zhou et al., 2017). Chen prepared a vertically oriented quasi-2D (BA)_2_(MA)_n−1_Pb_n_I_3n+1_ (*n* = 3, 4) HPs film by one-step spin coating using ammonium thiocyanate (SCN) as an additive. The study found that the addition of SCN was beneficial for the formation of the film, and obtaining a better out-of-plane orientation. The ITO/PEDOT structure perovskite SC showed the PCE of 6.82% (*n* = 3) and 8.79% (Zhang et al., 2017a).

Doping is also a method to improve the PCE of PDs SCs by changing the charge transfer, crystallization quality and band gap properties of thin films. Zhang et al. doped Cs^+^ into quasi-2D perovskite (BA)_2_ (MA)_3_Pb_4_I_13_, and resulted in the increase of the grain size and carrier mobility, the reduced defect density and the improved surface quality (Tsai et al., 2016). Finally, the PCE of the prepared perovskite SCs reached 13.7% with good stability, and the performance of the Cs^+^ doped devices stored in air with 30% humidity for 1,400 h was only reduced by 10% (Zhang et al., 2017b).

Compared with the 3D counterparts, 2D perovskite materials are more stable. Although some issues, such as low PCE and the preparation of the pure phase materials are existed. However, these problems can be solved by using different spacer cations, and the value of n can be controlled so that high PCE devices can be prepared. In addition, for large n value, hot casting method is a good method to obtain high-quality perovskite films through precursor engineering. From the Table 1, the value of n increases, and the relative value of PCE also increases. However, for the value of *n*, an optimum value is necessary for obtaining the best device performance. In the later stage, the new spacer cations may be a good choice and the use of the Cs ions may also improve the material properties.

**TABLE 1:** Performance comparison of different 2D materials.

2D perovskite as absorbers	Spacer cation	Perovskite	Voc [V]	Jsc [mA/cm^2^]	FF	PCE [%]	Reference
	PEA	(PEA)_2_(MA)_2_Pb_2_I_10_	1.18	6.72	0.60	4.73	32
Hot-casting	BA	(BA)_2_(MA)_2_Pb_3_I_10_	0.93	9.42	0.46	4.02	33
		(BA)_2_(MA)_3_Pb_4_I_13_	1.01	16.76	0.74	12.52	34
		(BA)_2_(MA)_4_Pb_5_I_16_	0.98	15.5	0.65	10.0	34
Add SCN one-step spin coating	BA	(BA)_2_(MA)_2_Pb_3_I_10_	0.97	12.79	0.55	6.89	38
		(BA)_2_(MA)_3_Pb_4_I_13_	0.98	14.71	0.61	8.79	38
		(BA)_2_(MA)_4_Pb_5_I_16_	1.11	15.01	0.67	11.01	38
RT one-step spin coating	iso-BA	(iso-BA)_2_Pb_4_I_13_	1.14	14.87	0.52	8.82	36
Doping Cs	BA	(BA)_2_[Cs_0.05_(MA)_0.95_]_3_Pb_4_I_13_	1.08	19.95	0.63	13.68	35
Introducing FA	BA	BA_2_(MA_0.8_FA_0.2_)Pb_4_I_13_	0.99	18.12	0.71	12.81	37

#### 2D/3D Mixed HPs as Light Absorbing Materials

The 2D/3D mixed HPs has been extensively studied by researchers in recent years because of its high stability and high photoelectric performance.

By adjusting the proportion of BA and FA/Cs in the BA_x_(FA_0.83_Cs_0.17_)_1-x_Pb(I_y_Br_1-y_)_3_ HPs film, Snaith reported a crystal perpendicular to the plane of the film between 3D grains. The XRD analysis revealed that the crystal was a 2D layered perovskite. The results show that the growth of 3D perovskite crystals is limited by 2D perovskite crystals due to the good lattice matching of 3D perovskite and 2D perovskite in the precursor solution after adding BA. Thus, the [100] preferred growth orientation aligned parallel to the film normal is exhibited. The average PCE of the optimized perovskite thin film solar cells was 17.5% (Eg = 1.61 eV) and 15.8% (Eg = 1.72 eV), respectively; the highest PCE of the device was 20.6%. In addition, the device has good stability. After 1,000 h of storage in an air environment, the PCE of the device can still maintain more than 80% of its initial value (Wang et al., 2017).

**FIGURE 5:**
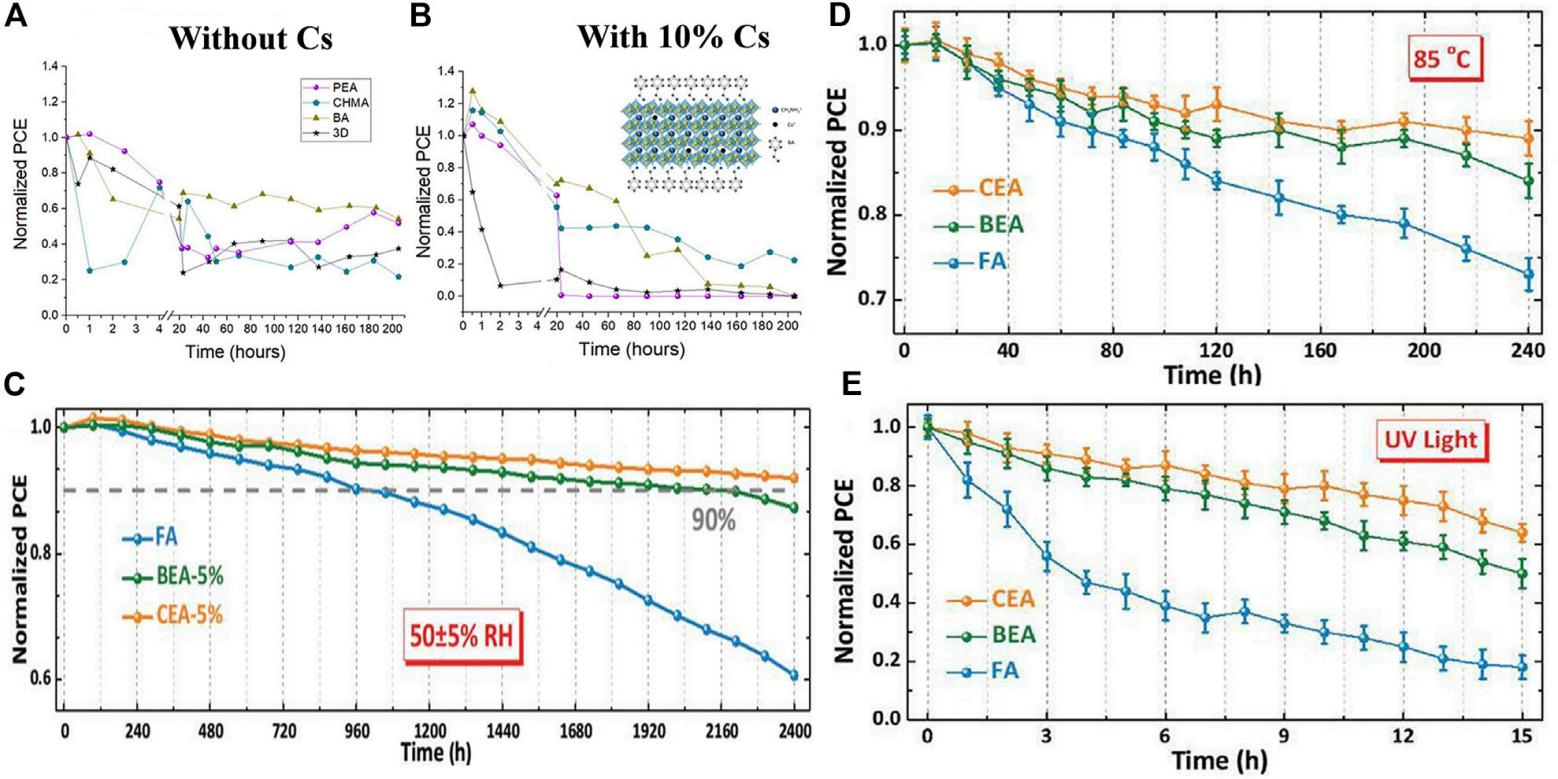
**(A)** Stability testing of 2D/3D HPs and 3D SCs based on different spacers at AM = 1.5G continuous illumination and ambient humidity of 30–50%; Reproduced with permission from Iagher and Etgar, 2018. Copyright 2018 ACS Energy Lettes. **(B)** Stability testing of unpackaged 2D/3D HPs and 3D perovskite SCs after storage for different periods of time in an air environment with a humidity of 50 ± 5%; Normalized PCE variation curves of FA, CEA-5%, and BEA-5% PSCs when exposed to **(C)** 85°C and **(D)** continuous UV irradiation. Reproduced with permission from Liu et al., 2019. Copyright 2019 Advanced Functional Materials.

Iagher and coauthors studied the effects of different spacer molecules (PEA/BA/CHMA (Cyclohexyl methacrylate)/3D) and Cs-doped on 2D/3D HPs structures (Iagher and Etgar, 2018). They found that when Cs ions were added to 2D/3D HPs, their photovoltaic performance was improved. The spacer molecule was PEA and the best photovoltaic performance was obtained when 10% Cs was added: the PCE, the Voc, the Jsc and the FF was 14.33%, 0.92 V, 25.5 mA/cm^2^ and 61%, respectively. On the other hand, the stability of SCs decreased with the addition of cesium (Figures 5A,B). Compared with pure MA SCs, the SCs based on (MA, Cs) mixed cations show relatively poor stability. The reason may be that the perovskite structure is deformed due to the mixing of two cations with different ion radii. This similar result has also been reported previously (Mitzi et al., 2001; Xu et al., 2003), and the increase in strain and distortion accelerated the degradation process. CHMA-based spacer molecules with mixed cations exhibited better stability and could be explained by the nature of the aliphatic ring, helping to promote polar NH_3_^+^ functional groups, thereby minimizing deformation of the inorganic skeleton due to the addition of Cs. Spacer molecules will not greatly affect the photovoltaic performance of the SCs. However, due to different spacer molecules, the stability of SCs will be different. In general, aromatic spacer molecules (BA) exhibit better stability than ring spacer molecules (PEA) (Iagher and Etgar, 2018).

Chen reported a 2D/3D HPs nano-structure by doping NAP into CH_3_NH_3_Sn_0.5_Pb_0.5_I_x_Cl_3-x_ perovskite, in which 2D components are seamlessly connected with 3D components to form 2D/3D interfaces (Chen et al., 2018a). This was the first report on the structure of the hybrid 2D/3D HPs. In addition, based on the 2D/3D HPs structure, a low band gap perovskite SC was successfully prepared with PCE greater than 12%. When 9 mM NAP was added, the device showed the best optoelectronic performance the FF of 69.5%, with the Voc of 0.71 V, the Jsc of 27.1 mA/cm^2^, and the PCE of 13.4%. More encouragingly, the device shows good stability and is one of the most stable tin-based perovskite SCs reported so far.

The intrinsic hydrophobicity of the ammonium salt has a great influence on the long-time humidity stability of the SCs. By introducing a long-chain alkyl ammonium salt, the hydrophobicity increased, but the charge transport between the layers got bad, thereby limiting the improvement of the device efficiency. To solve the above problems, Liu introduced a short-chain ammonium salt containing a halogen functional group to effectively increase the hydrophobicity of the ammonium salt, which improves the device efficiency and the stability of the device, and finally obtaining efficient and stable 2D/3D HPs SCs. In the Cs/FA mixed cation 3D perovskite material, 2-bromoethylamine hydrobromide (BEA) and 2-chloroethylamine hydrochloride (CEA) were introduced to form 2D/3D HPs, respectively. When 5% CEA was introduced into the SCs, 20.08% of the PCE was obtained, which was significantly improved compared with 18.97% of the 3D perovskite device (Liu et al., 2019). In addition, the introduction of ammonium salts containing halogen functional groups greatly increased the humidity stability of the SCs. After the device was stored in air with humidity of 50 ± 5% for 2,400 h, the PCE of the 2D/3D HPs device could still be maintained at about 92% of the initial value, while the PCE of the 3D perovskite device was maintained at only about 60% of the initial value (Figure 5C). Furthermore, the 2D/3D perovskite device showed excellent thermal stability and ultraviolet light stability (Figures 5D,E).

From the Table 2, after the 2D and 3D perovskites are mixed, the PCE of the SCs has been greatly enhanced, and the device also shows the high stability for the mixed dimension materials. A small amount of 2D components can significantly improve device performance, and it can also act as a barrier to prevent material from being eroded and to control crystal growth orientation. Doping or spacer cation concentration also shows a large impact on the material, so controlling the concentration is the most critical issue which can be solved by a concentration gradient and the best device performance will be obtained.

**TABLE 2:** Performance comparison of 2D/3D as light absorbing materials.

2D/3D perovskite as absorbers	Spacer cation	Perovskite	Voc [V]	Jsc [mA/cm^2^]	FF	PCE [%]	Reference
	BA	BA_0.05_(FA_0.83_Cs_0.17_)_0.95_Pb(I_0.8_Br_0.2_)_3_	1.14	22.7	0.80	20.6	45
	BA	BA_0.05_(FA_0.83_Cs_0.17_)_0.95_Pb(I_0.6_Br_0.4_)_3_	1.18	19.8	0.73	17.2	45
Doping10%Cs	PEA	(PEA)_2_MA_39_Pb_40_I_121_/MAPbI_3_	0.92	25.5	0.61	14.33	41
Doping10%Cs	BA	(BA)_2_MA_39_Pb_40_I_121_/MAPbI_3_	0.93	22.4	0.53	11.10	41
Doping10%Cs	CHMA	(CHMA)_2_MA_39_Pb_40_I_121_/MAPbI_3_	0.90	23.6	0.59	12.58	41
Add 9 mlNAP	NAP	CH_3_NH_3_Sn_0.5_Pb_0.5_I_x_Cl_3-x_	0.71	27.1	0.7	13.4	44
5%CEA	CEA	[(CEA)_2_PbX_4_]_x_ [(Cs_0.1_FA_0.9_)Pb(I_0.9_Br_0.1_)_3_]_1-x_	1.1	22.77	0.79	20.08	5
5%BEA	BEA	[(BEA)_2_PbX_4_]_x_ [(Cs_0.1_FA_0.9_)Pb(I_0.9_Br_0.1_)_3_]_1-x_	1.11	22.53	0.79	19.8	5

#### 2D Layered Perovskite as Interface Engineering Layer

A thin 2D perovskite film can be introduced as an interfacial layer to the bottom or top of a 3D perovskite. This introduction usually depends on the self-assembly (Tong et al., 2018) and *in-situ* formation (Chen et al., 2018b) of 2D perovskite.

Li et al. used a 2D perovskite formed *in-situ* as an interfacial layer for the preparation of a stable and efficient MAPbX_3_ perovskite SCs (Yao et al., 2015). By inserting branched polyethyleneimine hydroiodide (PEI HI) on top of the hole transport layer (PEDOT: PSS), which helps to form a thin (PEI)_2_PbI_4_ layer during the deposition of MAPbX_3_ perovskite. Finally, they prepared HPs SC with PCE of more than 16 and 13.8% on rigid and flexible substrates, respectively.

**FIGURE 6:**
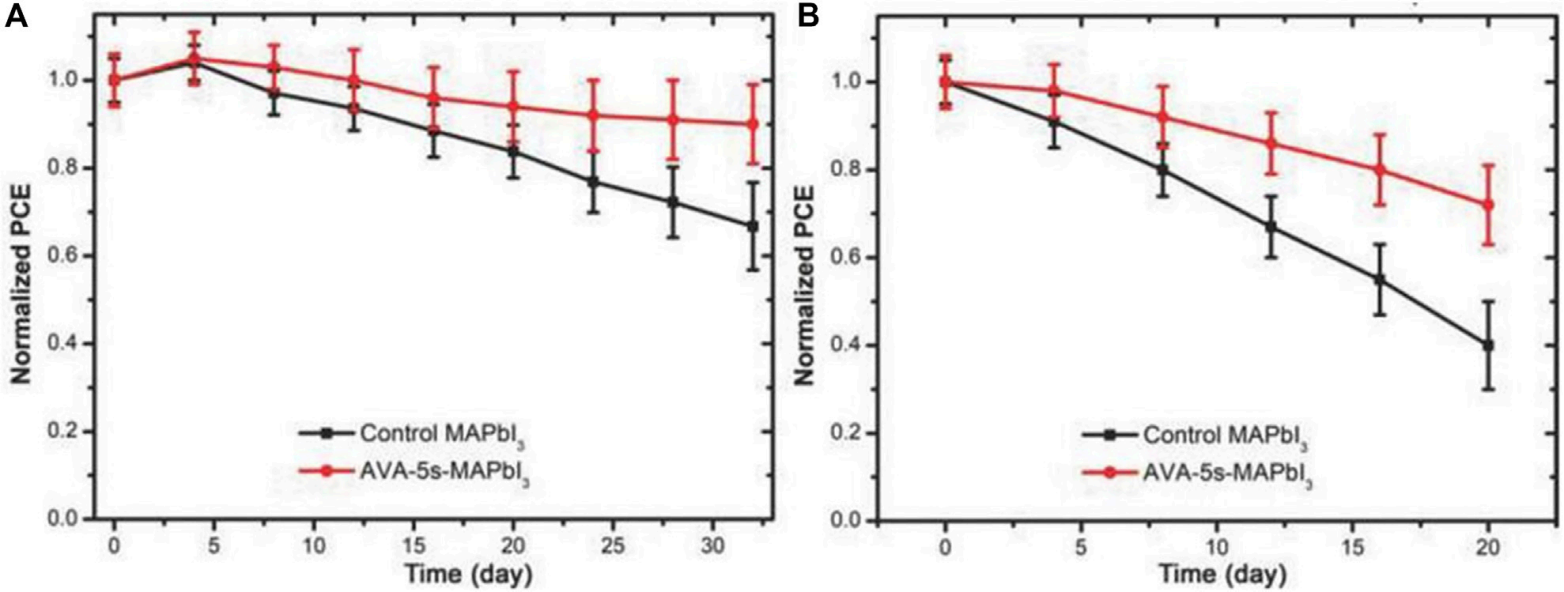
Stability test of untreated and rapidly annealed perovskite SCs after storage in **(A)** argon-filled glove boxes and **(B)** humidity of 40% for different days. Reproduced with permission from Ye et al., 2018. Copyright 2018 Advanced Functional Materials.

Ye et al. used an improved rapid annealing method on the surface of the 3D perovskite to release the organic halide CH_3_NH_3_I, leaving an inorganic Pb-I skeleton on the surface, and 5-aminopentanoic acid iodide was added by casting an additive solution on the annealed film ((HOOC(CH_2_)_4_NH_3_I, AVAI (5-aminovaleric acid iodide)) to form a 2D perovskite structure. This 2D/3D HPs manufacturing method provided a good solution for the manufacture of highly efficient and stable perovskite film SCs. When the annealing time is 5s, the PCE of the device reached 18%. More importantly, the stability of the device was improved (Figures 6A,B). When the device was stored in an argon-filled glove box for 32 days, the PCE of the 2D/3D HPs device fabricated by rapid annealing could still be maintained at about 90% of its initial value, while the PCE of the 3D perovskite device was only maintained at about 67% of its initial value. In addition, the PCE of 3D perovskite device decreased to 40% of its initial value after storing in 40% humidity air for 20 days, while the PCE of the 2D/3D HPs device could remain at 72% of its initial value. This 2D/3D HPs synthesis could be used to improve the humidity, heat, and oxygen stability for the perovskite film devices (Ye et al., 2018).

Lin et al. reported that after preparing polycrystalline MAPbI_3_ thin films by one-step spin-coating method, BA chlorobenzene (CB) solution and BAI isopropanol (IPA) solution were spin-coated on the 3D perovskite layer, respectively. Finally, the 2D perovskite thin layer was formed by self-assembly on the 3D perovskite polycrystalline film. This was a 2D/3D stacked structure, but the molecular structure of the 2D perovskite layer is different. The study found that the 2D layered film not only enhanced the stability of the perovskite SC, but also reduced the defect density of the perovskite. It also increased the carrier lifetime and deactivated the defects of the perovskite surface, and finally a device with up to 19.56% PCE was obtained (Lin et al., 2018).

Yang deposited a 2D (PEA_2_Pb_2_I_4_) perovskite on a bulk 3D perovskite (MAPbI_3_) by solvent engineering and formed a 3D-2D gradient interface. This method is different from the traditional solvent engineering method: PEAI/toluene solution is used to replace pure toluene. The presence of the 3D-2D gradient interface improved the interface level, resulting in a device with a PCE of 19.89% and an ultra-high Voc of 1.17 V. More than that, the existence of the gradient interface as a self-wrapping layer inhibited the diffusion of internal cross-layer ions, and reduced the deterioration of the active layer in the surrounding environment (Bai et al., 2017).

Compared with the 2D/3D mixed HPs as a light-absorbing material, adding 2D perovskite as an interface layer is simpler. Interestingly, the total thickness and n value of 2D HPS layer can be changed by adjusting the concentration and chemical composition of the solution, which also provides a variety of ways for controlled material engineering. In addition, self-assembly and *in-situ* formation of 2D perovskites are mainly distributed on the surface of the entire active layer, which guarantees all the advantages of 3D perovskite. From the Table 3, the similarity between the 2D and 3D perovskite structures ensures a well-matched interface between the 2D and 3D layers, providing a means for the preparation of high-efficiency perovskite SCs.

**TABLE 3:** Performance comparison of 2D as interface engineering layer.

2D as interface engineering layer	Spacer cation	Perovskite	Voc [V]	Jsc [mA/cm^2^]	FF	PCE [%]	Reference
	AVAI	(AVAI)_2_PbI_4_/MAPbI_3_	1.06	22.3	0.76	18.0	49
	BA	(BA)_2_PbI_4_/MAPbI_3_	1.11	22.49	0.784	19.56	50
	BAI	(BA)_2_(MA)_n-1_Pb_n_I_3n+1_/MAPbI_3_	1.09	22.59	0.766	18.85	50
	PEA	MAPbI_3_/(PEA)_2_PbI_4_	1.17	21.8	0.78	19.89	51

#### The Planar Heterojunction Materials

The planar heterojunction consists of two layers, the donor and the acceptor, which are a hole and electron transport type, respectively. Generally, the donor layer is a photosensitive material having a high light absorption coefficient, and the acceptor layer generally has a high electron mobility rate and its lowest unoccupied molecular orbital is much lower than the donor material. According to the difference of the organic semiconductor materials in the active layer, the heterojunction can be divided into a single mass junction, a planar heterojunction, a bulk heterojunction, etc. Among them, the planar heterojunction is the most common organic SC structure used in the past. Planar heterojunction perovskite SCs have two types: n-i-p type and p-i-n type. The n-i-p structure is directly developed on the basis of the n-i-type planar heterojunction perovskite SC, which is similar to the reverse device structure of the bulk heterojunction organic/polymer SC and the difference is the use of perovskite material as the active layer. The introduction of a hole transport layer between the perovskite layer and the metal electrode helps to achieve balanced collection of electrons and holes.

**FIGURE 7:**
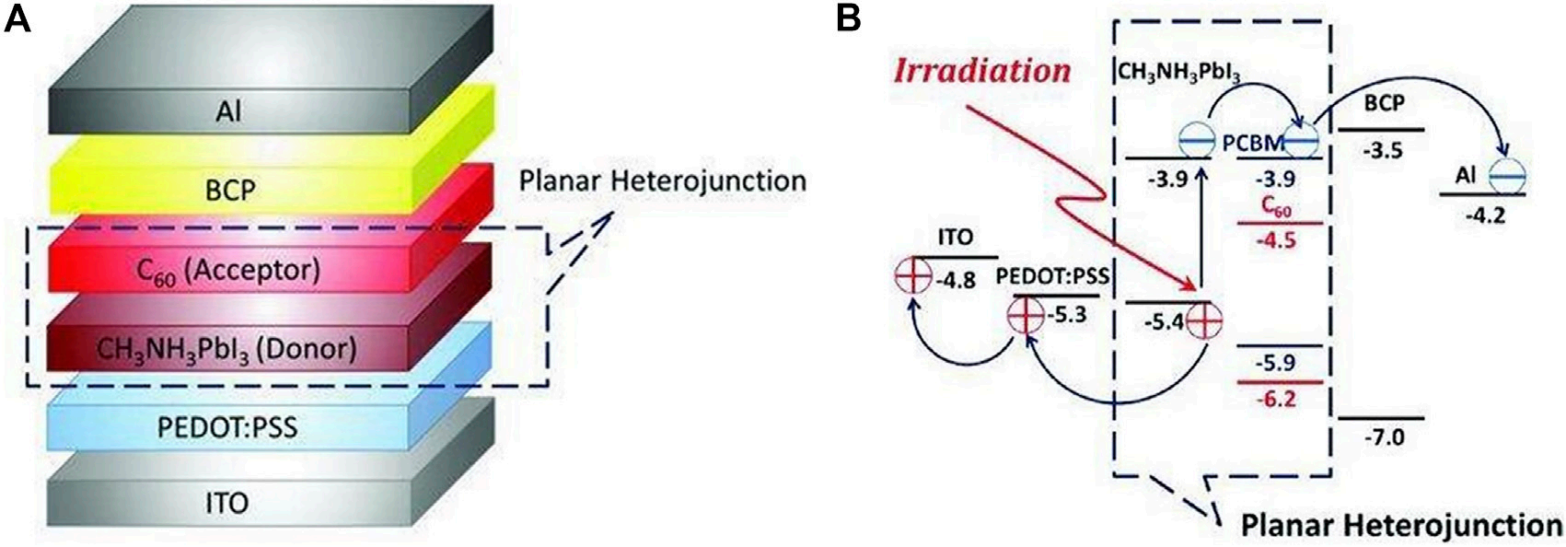
**(A)** Configuration of the hybrid solar cell. **(B)** Scheme of the energy levels of each layer in the device. Reproduced with permission from Jeng et al., 2013. Copyright 2013 Advanced Materials.

Snaith et al. used a dual source co-evaporation method to prepare a perovskite layer directly on the TiO_2_ dense layer and introduced spiro-OMeTAD as a hole transport layer to prepare the first n-i-p type planar heterojunction perovskite SCs (Liu et al., 2013). At the same time, Chen’s group presented a hybrid organic solar cell that used a glass/indium titanium oxide (ITO)/poly(3,4-ethyl-enedioxythiophene) poly(styrene-sulfonate) (PEDOT:PSS) substrate as the positive electrode, a PHJ of CH_3_NH_3_PbI_3_ perovskite/fullerene (C_60_) structure as the active layer, a thin bathocuproine (BCP) film as an exciton or hole-blocking layer (EBL or HBL), and an aluminum (Al) negative electrode (Jeng et al., 2013).The working principle was shown in Figures 7A,B. In Figure 7B, the LUMO and the highest occupied molecular orbital (HOMO) levels of CH_3_NH_3_PbI_3_ perovskite were –3.9 and –5.4 eV, respectively, and those of C_60_ were –4.5 and –6.2 eV. Under irradiation, excitons were generated by the absorption of light in the CH_3_NH_3_PbI_3_ perovskite layer. The oppositely charged holes and electrons in excitons were then separated at the donor–acceptor interface, as depicted in Figure 7B, and were transported by CH_3_NH_3_PbI_3_ perovskite and C_60_, respectively, resulting in the photovoltaic effect. Yang’s research group optimized the perovskite layer growth process and the electron transport layer to prepare a n-i-p-type planar heterojunction cell with FTO/PEIE/Y-TiO_2_/CH_3_NH_3_PbI_3_/spiro-OMeTAD/Au structure, which improved the photoelectric conversion efficiency to 19.3.% (Zhou et al., 2014).

Inspired by the n-i-type planar heterojunction structure, Guo et al. reported the p-i-n-plane heterojunction structure of polymer SCs as a reference to make fullerene derivatives ([6, 6]-phenyl-C61-butyric acid methyl ester, PC_60_BM) as the electron transport layer for the first time, and the PEDOT:PSS was acted as a hole transport layer to prepare the first p-i-n-type perovskite and fullerene planar heterojunction SC with ITO/PEDOT:PSS/CH_3_NH_3_PbI_3_/PC_60_BM/BCP/Al’s the structure (Jeng et al., 2013). The generated excitons are separated at the interface between the perovskite/hole transport layer and the perovskite/electron transport layer. The electrons are transferred from the conduction band of the perovskite layer to the LUMO level of PC_60_BM, and the holes are transferred through the perovskite layer to the PEDOT:PSS layer at the PEDOT:PSS/CH_3_NH_3_PbI_3_ interface, which is finally collected by ITO. However, in the preparation of the p-i-n-type perovskite cell, It is difficult to prepare perovskite transport layer with uniform thickness and flat film on PEDOT:PSS surface by one-step spin coating. Therefore, the thickness obtained perovskite layer is only 20–30 nm and far from being fully absorbed by sunlight. Therefore, the efficiency of the cell is very low, only 3.9%. When the thickness of perovskite film reaches 100 nm, the PCE of the device can be increased to 7.41% (Sun et al., 2014). Due to the photoinduced attenuation caused by ultraviolet light, the TiO_2_ electron transport layer is not very good for the stable p-i-n-type planar heterojunction perovskite cell. In addition, each carrier transport layer can be prepared at low temperature, which is beneficial to the preparation of low-cost large-area flexible perovskite SCs. After continuous optimization from the battery structure and the preparation process, the PCE of the p-i-n planar heterojunction SC had exceeded 12% (Chiang et al., 2014; Kim et al., 2014; Malinkiewicz et al., 2014; Seo et al., 2014; Xiao et al., 2014b; Wang et al., 2014; Zhang et al., 2014). By further optimization, the device efficiency has been increased to above 24% (NREL, 2020).

The perovskite material CH_3_NH_3_PbI_3_ has high carrier mobility, lower exciton binding energy, wide absorption spectrum and high light absorption coefficient, which allows it to enough absorb sunlight and reduce the energy loss in the photoelectric conversion process. CH_3_NH_3_PbI_3_ is easily soluble in polar solvents, so it is easily decomposed in a humid environment. Even after being packaged, the cell decays quickly. Secondly, such perovskite materials’ thermal stability is not good. In addition, the electrode material is not the same, which will result in the device PCE with a large gap, so we should consider which material to use as the electron and the hole transport layer before preparing the complete device. Furthermore, there are many methods for fabricating heterojunction materials such as one-step method, two-step method, and vapor deposition method, etc., and there are advantages and disadvantages of various methods, so which method to be chosen depends on the experiment.

### Research Progress in Light-Emitting Diodes

The light-emitting diode is a device that combines electrons and holes injected in a semiconductor material to realize radiation (Era et al., 1994). For LEDs, the performance parameters mainly include: external quantum efficiency (EQE), photoluminescence quantum yield (PLQY), brightness or maximum brightness, and current efficiency (CE) (Kim et al., 2005).

Era prepared a 2D (C_6_H_5_C_2_H_4_NH_3_)_2_PbI_4_ perovskite material to assemble an electroluminescent device (Era et al., 1994). The device needs to be illuminated under liquid nitrogen conditions with a high turn-on voltage and a low quantum efficiency. Yuan prepared a PEA_2_(CH_3_NH_3_)_n-1_Pb_n_I_3n+1_ 2D perovskite material by using a larger C_6_H_5_CH_2_CH_2_NH^+3^ (PEA) as a cationic moiety (Yuan et al., 2016). At low voltages, this multi-component multilayer perovskite material exhibited an EQE of 8.8% and a lumen efficiency of 80 W sr^−1^ m^−2^ because the quantum well structure of the 2D material could promote carrier reflow, further promoted carrier recombination, resulting in the higher efficiency. Ling prepared high luminescence crystalline CH_3_NH_3_PbBr_3_ nanosheets with a quantum yield of up to 85%. The maximum effective electroluminescence was 10,590 cd m^−2^ (Ling et al., 2016). Wang chose the organic cation with larger L-site size to reduce the exciton diffusion length and increase the exciton binding energy, and improve the device based on the L_2_MX_4_ structure of 2D perovskite’s luminous efficiency (Wang et al., 2016).

**FIGURE 8:**
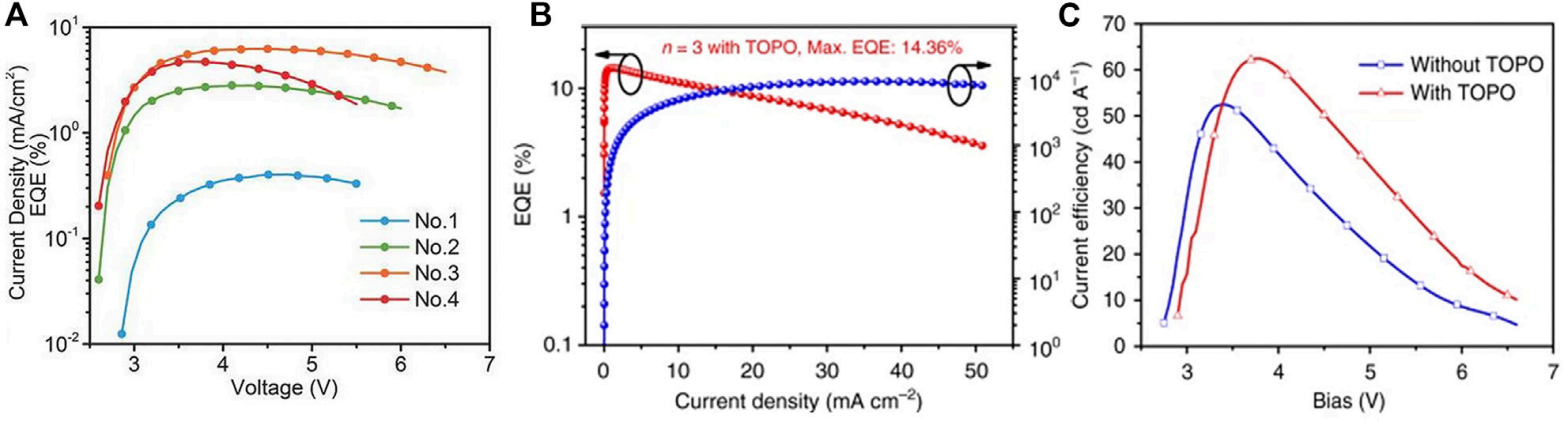
**(A)** The relationship between EQE and voltage based on BA_2_Cs_n-1_Pb_n_I_3n+1_device; Reproduced and permission from ref. 68. Copyright 2018 Advanced Materials. **(B)** the EQE of with TOPO passivate PEA_2_(FAPbBr_3_)_n-1_PbBr_4_ (*n* = 3) device; **(C)** the relationship between current efficiency and voltage of PEA_2_(FAPbBr_3_)_n-1_PbBr_4_ (*n* = 3) device without TOPO layer and with TOPO layer. Reproduced withpermission from Yang et al., 2018a. Copyright 2018 Nature Communications.

Xiao et al. reported a nanoscale microcrystalline perovskite LED by using the large ammonium halide as a surfactant added to the perovskite precursor solution (Xiao et al., 2017), which limited the growth of perovskite grains during the film formation process. A microcrystalline film with a size as small as 10 nm and a surface roughness of less than 1 nm was formed. The nanoscale perovskite grains combined with long-chain organic cations to obtain a highly efficient luminescent layer. The prepared 2D perovskite BA_2_MA_n-1_Pb_n_X_3n+1_ LED achieved an EQE of 10.4% (X = I) and 9.3% (X = Br) and didn’t degrade after 240 days in a nitrogen atmosphere. In the same year, Huang Wei’s team achieved high-performance red LED devices by introducing inorganic Cs into the multi-quantum well (MQW) 2D layered perovskite (NMA: N-Methyl-DL-Aspartic acid) (NMA)_2_Pb_n_I_3n+1_ (Zhang et al., 2017c). The MQW structure helped to form a cubic phase CsPbI_3_ perovskite at low temperatures, enabling Cs-based MQW to provide pure and stable red electroluminescence. Subsequently, the MaBiwu’s team of Florida State University of the United States used a composite film of quasi-2D perovskite material BA_2_Cs_n-1_Pb_n_I_3n+1_ and polyethylene oxide PEO as the luminescent layer to obtain an efficient and stable color adjustable from red to deep red (Tian et al., 2018).The LED showed a higher PLQE than the device with a quasi-2D perovskite luminescent layer alone, and the brightness and EQE reached 1,392 cd m^−2^ and 6.23%, respectively (Figure 8A). The Youjingbi’s team obtained green light devices with current efficiency and EQE up to 62.4 cd A^−1^ and 14.36%, respectively, by adjusting the composition and surface passivation of the luminescent layer materials used in the device (Figures 8B,C). For PEA_2_(FAPbBr_3_)_n-1_PbBr_4_, it was found that the device had the strongest luminescence when *n* = 3, and the LED with superior performance was obtained by passivating the surface of the quasi-2D perovskite film with trioctylphosphine oxide (TOPO) (Yang et al., 2018a). However, in quasi-2D perovskites, a balance existed between losing electrical conductivity and obtaining advantages including the exciton onfine-ment, enhancement of film quality, and reduced trap density (Byun et al., 2016). In the same year, Tsai et al. also reported that pure-phase 2D BA_2_MA_n-1_Pb_n_I_3n+1_ LEDs with adjustable color and high efficiency and stability could achieve the radiation efficiency of 35 W Sr^−1^ cm^−2^ and the ultra-low open voltage of 1 V at 744 nm by using vertically oriented thin films to promote charge injection and transmission. Tests showed that phase purity was closely related to stability, and pure phase 2D perovskite devices exhibited greater stability over 14 h compared to that of the 3D perovskites (Tsai et al., 2018).

Radiation recombination occurs rapidly in 2D perovskite materials, which makes it an effective emitter layer for high efficiency perovskite LED (Wang et al., 2016; Yuan et al., 2016; Quan et al., 2017; Si et al., 2017; Xiao et al., 2017). However, due to the quantum confinement effect that increases electron-hole recombination and promotes radiation emission, the LED based on 2D perovskite nanosheets exhibits higher electroluminescence efficiency. As we all know, the structure of 2D nanosheets (<*n*>=1) is unfavorable to the luminescence efficiency, because of the possible excitons’s thermal quenching at room temperature. Yang et al. developed a simple strategy to inhibit the NMA_2_PbBr_4_’s (<*n*>=1) growth nanosheets by meticulously adjusting formazan bromide (FABr) and (NMABr)’s the precursor ratios (Wu et al., 2018). When the NMABr ratio is less than 60%, the photoluminescence quantum yield (PLQY) increases due to the size limiting effect. Badly, the NMA_2_PbBr_4_ component/3D perovskite in 2D also increases with increasing NMABr ratio, resulting poor EL efficiency. The addition of FABr can provide excessive control on the growth inhibition of NMA_2_PbBr_4_ in 2D/3D perovskite. The dense and uniform perovskite film with low content of NMA_2_PbBr_4_ can achieve ∼61% PLQY. From these characteristics, the green perovskite LED produces a current efficiency of 46.8 cd A^−1^ and an EQE of 14.9%.

**FIGURE 9:**
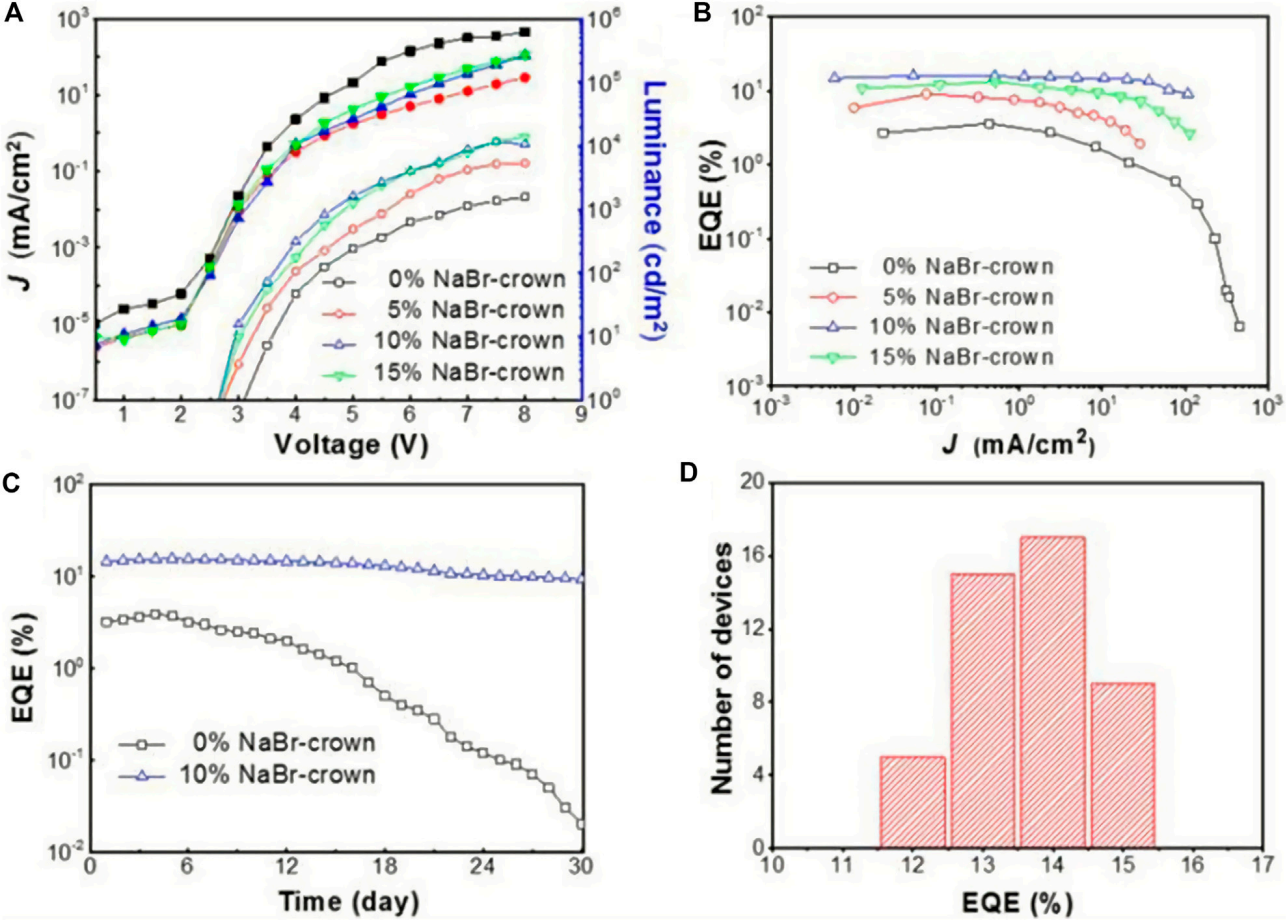
**(A)** The relationship between current density-voltage and luminance-voltage of 2D/3D perovskite devices with different NaBr molar ratios; **(B)** the relationship between EQE and current density of 2D/3D perovskite devices with different NaBr molar ratios; **(C)** the relationship between EQE and storage days of 2D/3D perovskite devices without NaBr and with NaBr doping; **(D)** EQE statistical distribution of 20% NaBr doped 2D/3D HPs devices. Reproduced and permission from Wu et al., 2019. Copyright 2019 ACS Nano.

Wu et al. added sodium bromide (NaBr) to CsPbBr_3_ to form 2D-3D lead halide perovskites, and the PLQY of the LEDs was significantly improved (Wu et al., 2019). This 2D-3D perovskite exhibited a compact and uniform film, thus achieving up to 15.9% EQE and excellent stability. It was found that the molar ratio of NaBr significantly affected the PLQY of the perovskite film, which was consistent with the 2D perovskite with organic spacers. As the molar ratio of NaBr increases, PLQY increases due to the enhanced dielectric limitation, and the size distribution decreases (Kumagai and Takagahara, 1989). The 2D/3D perovskite film with 15% NaBr produced up to 51% PLQY, but as the NaBr molar ratio increased further, PLQY began to decrease, probably due to less composition of luminescent materials and the existence of ultra-thin Na_2_Cs_n-1_Pb_n_Br_3n+1_ (*n* < 2) plates. A comparison of Figures 9A–D shows that 10% NaBr will optimize device performance with an EQE of 15.9%. In addition, the stability of the device is improved after the addition of NaBr. At the same time, Ning’s team reported the preparation of nanocomposites with homogeneous nanoscale quasi-2D PEA_2_Cs_n−1_PbnBr_3n+1_ perovskite quantum well. They then inserted inorganic crystalline Cs_4_PbBr_6_ to separate PEA_2_Cs_n−1_PbnBr_3n+1_ perovskite. By reducing carriers’ diffusion path, and forming cascading band structure alignment, they successfully increased the carriers’ concentration at the emitting zone, leading to much improved luminescence quantum yield. They fabricated LEDs based on this nanocomposite material and studied the effect of the insertion of Cs_4_PbBr_6_. Compared with the materials without Cs_4_PbBr_6_, after adding 20% of Cs_4_PbBr_6_, the materials’ maximum luminance increased from 28 to 3,259 Cd/m^2^, and the EQE increased from 0.6 to 4.51% (Shang et al., 2018).

From the Table 4, 2D or 2D/3D hybrid perovskite LED devices behave many advantages, such as high brightness illumination, high PLQY, etc., and the performance of the device is better than their 3D counterparts. The main reason is that the exciton binding energy of the 2D or 2D/3D hybrid perovskites is large, the PLQE is high, and the radiation recombination can be effectively performed. However, the experiment shows that pure 2D will have low luminous efficiency and other issues, so the quasi-2D and 2D/3D mixing perovskite are more attractive for better device performance. In addition, unfortunately the low n value of the 2D composition in the mixed material should not be excessive, which will result in a decrease in PLQY. Therefore, when preparing a 2D/3D mixed material, it is necessary to control the growth of the 2D component.

**TABLE 4:** Performance comparison of various LED.

Perovskite	PL/EL (peak position)	PLQY [%]	EQE [%]	CE [cd/A]	PE [Lm/W]	Reference
BAI:MAPbI_3_(20:100)	750/748	1.9	10.4	0.09	0.10	66
BABr:MAPbI_3_(20:100)	516/513	7.00	9.3	17.1	13.0	66
(NWA)_2_CsPb_2_I_6_ClMQWs	--/688	--	3.7	--	--	67
BA_2_Cs_n-1_Pb_n_I_3n+1_/PEO	--/680	--	6.23	1.74	1.37	68
(PEA)_2_(FAPbBr_3_)_2_PbBr_4_	532/--	57.3	12.12	52.51	--	69
(PEA)_2_(FAPbBr_3_)_2_PbBr_4_(with TOPO)	532/--	73.8	14.36	62.43	--	69
CsPbBr_3_+60%NMABr(FABr)	--/514	61	14.9	46.8	--	76
CsPbBr_3_+10%NaBr	--/518	40	15.9	50.3	45.1	77

### Research Progress in Photodetectors

Photodetectors are semiconductor devices which convert optical signals into electrical signals and play an important part in imaging systems, optical communications, biosensing, and environmental monitoring (Gong et al., 2009; Dong et al., 2011; Zhao, 2011; Li Chong et al., 2014; Büchele et al., 2015; Yang Hua et al., 2015; Wang Yan and Zhang Rui, 2016; Yan Peiqin et al., 2017; Tong et al., 2017; Yang et al., 2018b; Zhou et al., 2018b; Zhang et al., 2020a; Wu et al., 2020; Pan et al., 2020; Loi et al., 2020; Song et al., 2021; Pan et al., 2021). In general, the working principle of a photodetector includes three processes: 1) absorption of incident light to generate carriers; 2) carrier migration; 3) the collection of the carriers to generate current signals. The device-performance parameters mainly cover responsivity (R), detectivity (D*) external quantum efficiency (EQE), light/dark current ratio, photoconductive gain (G) and response time (Gao Xiuyun et al., 2018; Wang Jiaojiao et al., 2018). At present, the photodetector based on 2D perovskite materials has attracted great attention due to the extraordinary characteristics of the 2D perovskites, and some reports are summarized below.

2D layered perovskites behave superior carrier transport properties compared to pure organic polymers, such as they are easier to form films and can obtain uniform and dense films by one-step process without the need for high temperature annealing (Jeon et al., 2014; Zhang et al., 2016). In addition, the unique structure and properties of 2D layered perovskites, such as quantum confinement effects and bandgap tunability, make them tunable photo responses. As a direct bandgap semiconductor, the 2D perovskite material has a high absorption coefficient, a high quantum yield, and a continuous adjustable band gap in the visible spectrum, so it shows a high application value in the photodetector (An et al., 2013; Tan et al., 2016; Li et al., 2020; Jing et al., 2020; Wang et al., 2021; Li et al., 2021). The organic composition of the 2D layered perovskite provides a structural diversity compared to conventional 3D perovskites. The inherent instability of traditional three-dimensional (3D) organic–inorganic hybrid perovskites limited their further application in devices (Liang et al., 2017; Liu et al., 2020). As mentioned above, 2D layered perovskite materials have made breakthroughs in photovoltaic devices and electroluminescence, so it is expected to develop a new generation of low-cost and high-performance photodetectors based on 2D layered perovskite materials (Zhang et al., 2020b; Xia et al., 2020).

**FIGURE 10:**
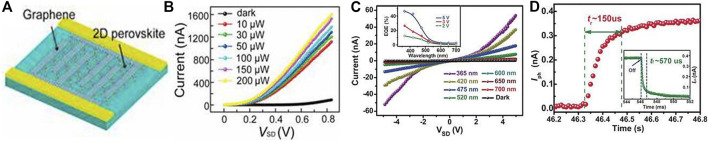
**(A)** The PD based on BA_2_PbBr_4_’s schematic diagram (Tan et al., 2016); **(B)** the relationship between the current and voltage of the photodetector without illumination and under different illumination (Tan et al., 2016); Reproduced with permission from Tan et al., 2016. Copyright 2016 Journal of the American Chemical Society. **(C)** the relationship between current and voltage of photodetectors based on BA_2_MA_2_Pb_3_Br_10_ without illumination and under different wavelength laser irradiation; **(D)** the response time of photodetectors. Reproduced with permission from Li et al., 2017. Copyright 2017 Angewandte Chemie International Edition.

Ahmad et al. prepared a photodetector based on pure 2D perovskite material (C_6_H_9_C_2_H_4_NH_3_)_2_PbI_4_ (Ahmad et al., 2015). It was found that the addition of hole and electron transport layers can greatly increase photocurrent. In addition, the cooperation of TiO_2_ nanoparticles further increases the photocurrent, and ultimately achieving 10% EQE at a wavelength of 508 nm. Zhou et al. prepared photodetectors with 2D layered perovskite materials BA_2_MA_n-1_Pb_n_I_3n+1_ (*n* = 1, 2, 3) with different n values and the device structure (Yao et al., 2016). Since different n values correspond to different band gaps, optical response at different wavelengths can be achieved with a response time of the order of milliseconds. Under white light with a bias voltage of 30 V and a light intensity of 3.0 mW/cm^2^, the responsivity of the devices corresponding to *n* = 1, 2 and 3 is 3.00, 7.31 and 12.78 mA/W, respectively. In the same year, Liu et al. synthesized a single crystal 2D perovskite BA_2_PbBr_4_ nano-thick sheet photodetector and the device structure is shown in Figure 10A (Tan et al., 2016). The electrode is a graphene interdigital electrode, which realizes the R of ∼2100 A/W, a dark current of ∼10^–10^ and the light/dark current ratio of ∼10^3^. Luo et al. studied the ferroelectric properties of 2D perovskite BA_2_MA_2_Pb_3_Br_10_ and the performance of its single crystal photodetectors. As shown in Figures 10C,D, very low dark current (∼10^–12^ A), high light-dark current ratio (∼2.5 × 10^3^) and fast response time (∼150 μs) were obtained) (Li et al., 2017).

Liu used BN as a buffer layer to grow a 2D CH_3_NH_3_PbI_3_ nanodisk array on a Si/SiO_2_ substrate to realize a phototransistor (Liu et al., 2016). At different laser intensities at 405 nm, the device’s the photocurrent-time curve shows a significant difference, which confirms the effectiveness of the material as a photo-controlled switch. At a bias of 1 V, the responsivity at 405 and 532 nm is approximately 22 and 12 A W^−1^, respectively. Under the influence of a bias voltage, the on/off ratio is about 10^2^, and the rise and fall times of the device are less than 20 and 40 ms, respectively. All-inorganic 2D perovskites have also demonstrated excellent performance as photodetectors. Song reported a 2D CsPbBr_3_ nanosheet flexible photodetector with atomic thickness. The device showed the on/off’s ratio is greater than 10^4^ and the R is 0.25 A W^−1^, which is superior to commercial silicon-based optoelectronics. In addition, the stability of devices was better than the PD based on MAPbI_3_ (Song et al., 2016). Zhou’s group introduced a pressure-assisted annealing strategy to effectively reduce the void density and lower the surface roughness. The self-powered all-inorganic CsPbBr_3_ perovskite microcrystal (MC) thin film PD showed high performance characteristics, the R and D* of up to 0.206 A W^−1^ and 7.23 × 10^12^ Jones, respectively. Besides, the on/off ratios of the devices are up to 10^6^, and the highest linear dynamic range (LDR) reaches 123.5 dB (Zhou et al., 2018a).

Wang et al. developed a perovskite photodiode based on a reduced-dimensional quasi-2D (Q-2D) perovskite structure. By incorporating phenylethyl ammonium iodide (PEAI) into MAPbI_3_, the crystallinity and environmental stability of perovskite were effectively enhanced (Lim et al., 2018). The dark current of Q-2D perovskite PD was 1.76 × 10^–7^ A/cm, the R was 0.53 A/W and the D* was 2.20 × 10^12^ Jones. In addition, even after 80 days of storage under environmental conditions, the current density of the Q-2D perovskite PD remains at about 76% of the initial value, which corresponds to only 15% of the initial value for the 3D perovskite PD. This excellent performance and stability is mainly due to the good crystallinity of Q-2D perovskite The stability of the Q-2D perovskite film was further confirmed by lifetime and atomic force microscopy test.

At present, 2D or 2D/3D HPs materials are less studied in the field of photodetectors as shown in the Table 5. In addition, exploring other 2D perovskite materials with excellent photoelectric performance and simple preparation process is also a direction in the future.

**TABLE 5:** Performance comparison of various PDs.

Perovskite	D (J)	Dark-current J_dark_ (A)	LDR (dB)	Responsivity (A/W)	Reference
(BA)_2_PbBr_4_	--	10^–10^	--	2,100	93
(BA)_2_ (MA)_2_Pb_3_Br_10_	3.6 × 10^10^	10^–12^	--	---	96
CsPbBr_3_(MCs)	7.23 × 10^12^	2.6 × 10^–7^	123.5	0.206	99
(PEA)_2_ (MA)_59_Pb_60_I_181_	2.2 × 10^12^	1.76 × 10^–7^	41	0.53	100
Cs(doping)-FAPbI_3_	2.7 × 10^13^	--	76.3	5.7	101
MA_0.7_FA_0.3_PbBr_3_	4.0 × 10^12^	--	100	0.51	102
MAPbI_3_	1 × 10^12^	--	120	0.55	103
CsPb_2_Br_5_-CsPbBr_3_	1.4 × 10^12^	4.24 × 10^–8^	128.6	0.11	105
MAPbBr_3_	6.59 × 10^11^	--	--	5,600	106
MAPbI_3_	4.16 × 10^12^	--	89	0.56	107
moiré-lattices MAPbI_3_	5.58 × 10^13^	--	71	15.62	108
CsPbBr_3_	4.2 × 10^12^	3.4 × 10^−8^	120.3	0.1	109
BDAPbI_4_	1.23 × 10^11^	--	150	0.927	110
Sr_2_Nb_3_O_10_	1.4 × 10^14^	2.1 × 10^–12^	--	1,214	111
(PEA)_2_ (MA)_n−1_Pb_n_I_3n+1_	2 × 10^12^	--	--	149	112
BA_2_PbBr_4_	10^12^	10^–14^	--	0.045	113
(PEA)_2_PbI_4_:Sn	6.77 × 10^13^	5.8 × 10^–13^	--	6.43	114

## Conclusion and Prospects

2D layered perovskite is formed by introducing large organic functional groups on the basis of 3D perovskite, which can be considered as nano-scale encapsulation of 3D perovskite, The stability of perovskite is improved. In addition, with the difference of n and R, many physical properties of 2D layered perovskite materials are also different, and due to their unique structure and photoelectric properties, the optoelectronic devices based on these 2D layered perovskites show good device performance.

In 2D perovskite-based SCs, large-volume organic cations hinder charge transport, resulting in poor carrier transport characteristics. The efficiency of the first reported 2D layered perovskite SC was only 4.73%, then the PCE of the devices was improved by adjusting the composition and film growth orientation, and the low band gap 2D layered perovskite SCs reached the PCE of 20.6%. In addition, many 2D/3D hybrid materials as light absorbing materials and 2D materials as interface engineering layer had been reported, their device performance not only achieved the high efficiency of 3D perovskite, but also showed many advantages, especially higher stability. Besides, The organic spacer cations that form 2D/3D mixed perovskite are mainly PEA and BA cations, and many other new cations such as iso-BA, TBA (Tribenzylamine), and IC_2_H_4_NH_3_ cations, were also demonstrated. These works encourage us to further explore acceptable trade-offs that will limit efficiency/poor stability (3D) to high stability and efficiency SCs. At the same time, it is still necessary to obtain more knowledge about the formation mechanism of 2D and 3D HPs, which will allow us to make high-performance 2D/3D HPs devices. In addition, a scalable deposition method should be demonstrated for 2D perovskites, as there are currently no reports of high-efficiency and large-area modules for these materials. Through material engineering, a wide variety of new 2D/3D perovskites can be manufactured, which will be very attractive to study their characteristics and performance in different aspects.

Besides, due to the high exciton binding energy of 2D laminar perovskite, it has a great advantage over 3D perovskite in the process of radiation recombination, and its components are more adjustable. Therefore, 2D laminar perovskite materials showed great applications in LEDs. In recent years, the EQE of the prepared LEDs has been continuously improved, and the emitting light colors have also been realized in purple, green and red. Although pure 2D perovskite LEDs displayed low luminescent efficiency, their 2D/3D mixed counterparts showed good stability and high EQE. In addition, how to construct novel structures to realize higher EQE needs to be further explored.

As application in photodetector, the 2D layered perovskite also shows strong interesting. These 2D layered perovskite photodetectors usually showed a metal-semiconductor-metal structure and achieved excellent performance. However, due to the layered structure, its self-powered performance with a p-i-n structure was not satisfied, so how to realize the high-performance photodetector with self-powered characteristic is a challenge.

Although optoelectronic devices based on 2D/3D HPs materials have showed excellent performance and good stability, The research on devices is very deep about operating mechanisms, material intrinsic properties, composite kinetics, and optical coupling properties are needed. In addition, the toxicity of the material has received extensive attention, and the lead component should be further removed to achieve the harmlessness of the material. Finally, further research is needed both in basic research and application fields.

## References

[B1] Ahmad S. Kanaujia P. K. Beeson H. J. Abate A. Deschler F. Credgington D. (2015). Strong Photocurrent from Two-Dimensional Excitons in Solution-Processed Stacked Perovskite Semiconductor Sheets. ACS Appl. Mater. Inter. 7, 25227–25236. 10.1021/acsami.5b07026 PMC4666456 26497547

[B2] Amat A. Mosconi E. Ronca E. Quarti C. Umari P. Nazeeruddin M. K. (2014). Cation-induced Band-gap Tuning in Organohalide Perovskites: Interplay of Spin-Orbit Coupling and Octahedra Tilting. Nano Lett. 14, 3608–3616. 10.1021/nl5012992 24797342

[B3] An X. Liu F. Jung Y. J. Kar S. (2013). Tunable Graphene-Silicon Heterojunctions for Ultrasensitive Photodetection. Nano Lett. 13, 909–916. 10.1021/nl303682j 23350824

[B4] Bai Y. Xiao S. Hu C. Zhang T. Meng X. Lin H. (2017). Dimensional Engineering of a Graded 3D-2D Halide Perovskite Interface Enables Ultrahigh V Oc Enhanced Stability in the P-I-N Photovoltaics. Adv. Energ. Mater. 7, 1701038. 10.1002/aenm.201701038

[B5] Baikie T. Fang Y. Kadro J. M. Schreyer M. Wei F. Mhaisalkar S. G. (2013). Synthesis and crystal Chemistry of the Hybrid Perovskite (CH3NH3)PbI3 for Solid-State Sensitised Solar Cell Applications. J. Mater. Chem. A. 1, 5628–5641. 10.1039/c3ta10518k

[B6] Büchele P. Richter M. Tedde S. F. Matt G. J. Ankah G. N. Fischer R. (2015). X-ray Imaging with Scintillator-Sensitized Hybrid Organic Photodetectors. Nat. Photon 9, 843–848. 10.1038/nphoton.2015.216

[B7] Byun J. Cho H. Wolf C. Jang M. Sadhanala A. Friend R. H. (2016). Efficient Visible Quasi-2D Perovskite Light-Emitting Diodes. Adv. Mater. 28, 7515–7520. 10.1002/adma.201601369 27334788

[B8] Cao D. H. Stoumpos C. C. Farha O. K. Hupp J. T. Kanatzidis M. G. (2015). 2D Homologous Perovskites as Light-Absorbing Materials for Solar Cell Applications. J. Am. Chem. Soc. 137, 7843–7850. 10.1021/jacs.5b03796 26020457

[B9] Chen P. Bai Y. Wang S. Lyu M. Yun J. H. Wang L. (2018). *In Situ* Growth of 2D Perovskite Capping Layer for Stable and Efficient Perovskite Solar Cells. Adv. Funct. Mater. 28, 1706923. 10.1002/adfm.201706923

[B10] Chen Y. Sun Y. Peng J. Zhang W. Su X. Zheng K. (2017). Tailoring Organic Cation of 2D Air-Stable Organometal Halide Perovskites for Highly Efficient Planar Solar Cells. Adv. Energ. Mater. 7, 1700162. 10.1002/aenm.201700162

[B11] Chen Z. Liu M. Li Z. Shi T. Yang Y. Yip H.-L. (2018). Stable Sn/Pb-Based Perovskite Solar Cells with a Coherent 2D/3D Interface. iScience 9, 337–346. 10.1016/j.isci.2018.11.003 30453163 PMC6240600

[B12] Chiang C.-H. Tseng Z.-L. Wu C.-G. (2014). Planar Heterojunction perovskite/PC71BM Solar Cells with Enhanced Open-Circuit Voltage via a (2/1)-step Spin-Coating Process. J. Mater. Chem. A. 2, 15897–15903. 10.1039/c4ta03674c

[B13] Collings I. E. Hill J. A. Cairns A. B. Cooper R. I. Thompson A. L. Parker J. E. (2016). Compositional Dependence of Anomalous thermal Expansion in Perovskite-like ABX3formates. Dalton Trans. 45, 4169–4178. 10.1039/c5dt03263f 26477747

[B14] Dong H. Zhu H. Meng Q. Gong X. Hu W. (2011). Organic Photoresponse Materials and Devices. Chem. Soc. Rev. 41, 1754–1808. 10.1039/c1cs15205j 22158983

[B15] Dou L. Wong A. B. Yu Y. Lai M. Kornienko N. Eaton S. W. (2015). Atomically Thin Two-Dimensional Organic-Inorganic Hybrid Perovskites. Science 349, 1518–1521. 10.1126/science.aac7660 26404831

[B16] Edri E. Kirmayer S. Cahen D. Hodes G. (2013). High Open-Circuit Voltage Solar Cells Based on Organic-Inorganic Lead Bromide Perovskite. J. Phys. Chem. Lett. 4, 897–902. 10.1021/jz400348q 26291353

[B17] Era M. S. TsutsuiMorimoto T. Saito S. (1994). Organic‐inorganic Heterostructure Electroluminescent Device Using a Layered Perovskite Semiconductor (C6H5C2H4NH3)2PbI4. Appl. Phys. Lett. 65, 676–678. 10.1063/1.112265

[B18] Fan Z. Sun K. Wang J. (2015). Perovskites for Photovoltaics: a Combined Review of Organic-Inorganic Halide Perovskites and Ferroelectric Oxide Perovskites. J. Mater. Chem. A. 3, 18809–18828. 10.1039/c5ta04235f

[B19] Galuskin E. V. Gazeev V. M. Armbruster T. Zadov A. E. Galuskina I. O. Pertsev N. N. (2008). Lakargiite CaZrO3: A New mineral of the Perovskite Group from the North Caucasus, Kabardino-Balkaria, Russia. Am. Mineral. 93, 1903–1910. 10.2138/am.2008.2900

[B20] Gao Xiuyun, Zhang Ye, Cui Yanxia, Liu Yanzhen, Li Guohui, Shi Linlin (2018). Research Progress in Organic Photomultiplication Photodetector 55, 070001. 10.3788/lop55.070001 PMC6165393 30208639

[B21] Gong X. Tong M. Xia Y. Cai W. Moon J. S. Cao Y. (2009). High-Detectivity Polymer Photodetectors with Spectral Response from 300 Nm to 1450 Nm. Science 325, 1665–1667. 10.1126/science.1176706 19679770

[B22] Han N. Yu T. Cui Y. X. (2018). Research Progress of 2D Layered Perovskite Materials and Their Applications. Laser Optoelectronics Prog. 8, 1006–4125. 10.3788/LOP56.070002

[B23] Iagher L. Etgar L. (2018). Effect of Cs on the Stability and Photovoltaic Performance of 2D/3D Perovskite-Based Solar Cells. ACS Energ. Lett. 3, 366–372. 10.1021/acsenergylett.7b01196

[B24] Jacobsson T. J. Pazoki M. Hagfeldt A. Edvinsson T. (2015). Goldschmidt's Rules and Strontium Replacement in Lead Halogen Perovskite Solar Cells: Theory and Preliminary Experiments on CH3NH3SrI3. J. Phys. Chem. C. 119, 25673–25683. 10.1021/acs.jpcc.5b06436

[B25] Jeng J.-Y. Chiang Y.-F. Lee M.-H. Peng S.-R. Guo T.-F. Chen P. (2013). CH3NH3PbI3Perovskite/Fullerene Planar-Heterojunction Hybrid Solar Cells. Adv. Mater. 25, 3727–3732. 10.1002/adma.201301327 23775589

[B26] Jeon N. J. Noh J. H. Kim Y. C. Yang W. S. Ryu S. Seok S. I. (2014). Solvent Engineering for High-Performance Inorganic-Organic Hybrid Perovskite Solar Cells. Nat. Mater 13, 897–903. 10.1038/nmat4014 24997740

[B27] Ji D. H. Zhang Y. Wang S. L. Li X. Zhang C. Zeng Z. (1994). First Principle Study of the B-Site Ordered Structure Perovskite BaFe_0.5_Nb_0.5_O_3_ . J. Hebei Univ. 4, 44–48.

[B28] Jin L. F. Zhang Y. T. Wang H. Y. Wang M. Song X. Yao J. (2014). Accelerated Aging of InGaAs PIN Photoelectric Detectors. Chin. J. Lasers. 8, 194–199. 10.3788/CJL201441.1008002

[B29] Jing H. Peng R. Ma R.-M. He J. Zhou Y. Yang Z. (2020). Flexible Ultrathin Single-Crystalline Perovskite Photodetector. Nano Lett. 20, 7144–7151. 10.1021/acs.nanolett.0c02468 32941049

[B30] Kim J. K. Luo H. Song C. Schubert E. F. Cho J. Park Y. (2005). Strongly Enhanced Phosphor Efficiency in GaInN White Light-Emitting Diodes Using Remote Phosphor Configuration and Diffuse Reflector Cup. Jpn. Soc. Appl. Phys. 5, 20–23. 10.1143/jjap.44.l649

[B31] Kim J. Kim G. Kim T. K. Kwon S. Back H. Lee J. (2014). Efficient Planar-Heterojunction Perovskite Solar Cells Achieved via Interfacial Modification of a Sol-Gel ZnO Electron Collection Layer. J. Mater. Chem. A. 2, 17291–17296. 10.1039/c4ta03954h

[B32] Koh T. M. Shanmugam V. Schlipf J. Oesinghaus L. Müller‐Buschbaum P. Ramakrishnan N. (2016). Nanostructuring Mixed‐Dimensional Perovskites: A Route toward Tunable, Efficient Photovoltaics. Adv. Mater. 28, 3653–3661. 10.1002/adma.201506141 26990287

[B33] Krishna A. Gottis S. Nazeeruddin M. K. Sauvage F. (2018). Mixed Dimensional 2D/3D Hybrid Perovskite Absorbers: The Future of Perovskite Solar Cells?. Adv. Funct. Mater. 29, 1806482. 10.1002/adfm.201806482

[B34] Kumagai M. Takagahara T. (1989). Excitonic and Nonlinear-Optical Properties of Dielectric Quantum-Well Structures. Phys. Rev. B. 40, 12359–12381. 10.1103/physrevb.40.12359 9991869

[B35] Lee M. M. Teuscher J. Miyasaka T. Murakami T. N. Snaith H. J. (2012). Efficient Hybrid Solar Cells Based on Meso-Superstructured Organometal Halide Perovskites. Science 338, 643–647. 10.1126/science.1228604 23042296

[B36] Li Chong, Zhang Dongliang, Xue Chunlai, Li Chuanbo, Cheng Buwen, Wang Qiming (2014). Progress in the Study of Si-Based Group IV Optoelectronic Devices (II)--Photodetectors 51, 110002. 10.3788/lop51.110002

[B37] Li J. Y. Han Z. Y. Gu Y. Yu D. Liu J. Hu D. (2021). Perovskite Single Crystals: Synthesis, Optoelectronic Properties, and Application. Adv. Funct. Mater. 3 (10), 2008684. 10.1002/adfm.202008684

[B38] Li L. Sun Z. Wang P. Hu W. Wang S. Ji C. (2017). Tailored Engineering of an Unusual (C4 H9 NH3 )2 (CH3 NH3 )2 Pb3 Br10 Two-Dimensional Multilayered Perovskite Ferroelectric for a High-Performance Photodetector. Angew. Chem. Int. Ed. 56, 12150–12154. 10.1002/anie.201705836 28719061

[B39] Li S. Y. Zhang Y. Yang W. Liu H. Fang X. (2020). 2D Perovskite Sr_2_Nb_3_O_10_ for High-Performance UV Photodetectors. Adv. Mater. 2 (22), 2003145. 10.1002/adma.201905443 31773828

[B40] Liang F.-X. Wang J.-Z. Zhang Z.-X. Wang Y.-Y. Gao Y. Luo L.-B. (2017). Broadband, Ultrafast, Self-Driven Photodetector Based on Cs-Doped FAPbI3 Perovskite Thin Film. Adv. Opt. Mater. 5, 1700654. 10.1002/adom.201700654

[B41] Liang Z. Tang K. Shao Q. Li G. Zeng S. Zheng H. (2008). Synthesis, crystal Structure, and Photocatalytic Activity of a New Two-Layer Ruddlesden-Popper Phase, Li2CaTa2O7. J. Solid State. Chem. 181, 964–970. 10.1016/j.jssc.2008.01.042

[B42] Lim J. W. Wang H. Choi C. H. (2018). Self-powered Reduced-Dimensionality Perovskite Photodiodes with Controlled Crystalline Phase and Improved Stability. Nano Energy 12, 761–770.

[B43] Lin D. Zhang T. Wang J. Long M. Xie F. Chen J. (2019). Stable and Scalable 3D-2D Planar Heterojunction Perovskite Solar Cells via Vapor Deposition. Nano Energy. 59, 619–625. 10.1016/j.nanoen.2019.03.014

[B44] Lin Y. Bai Y. Fang Y. Chen Z. Yang S. Zheng X. (2018). Enhanced Thermal Stability in Perovskite Solar Cells by Assembling 2D/3D Stacking Structures. J. Phys. Chem. Lett. 9, 654–658. 10.1021/acs.jpclett.7b02679 29350044

[B45] Ling Y. Yuan Z. Tian Y. Wang X. Wang J. C. Xin Y. (2016). Bright Light-Emitting Diodes Based on Organometal Halide Perovskite Nanoplatelets. Adv. Mater. 28, 305–311. 10.1002/adma.201503954 26572239

[B46] Liu G. Zheng H. Xu X. Xu S. Zhang X. Pan X. (2019). Introduction of Hydrophobic Ammonium Salts with Halogen Functional Groups for High‐Efficiency and Stable 2D/3D Perovskite Solar Cells. Adv. Funct. Mater. 29, 1807565. 10.1002/adfm.201807565

[B47] Liu J. Leng J. Wu K. Zhang J. Jin S. (2017). Observation of Internal Photoinduced Electron and Hole Separation in Hybrid Two-Dimentional Perovskite Films. J. Am. Chem. Soc. 139, 1432–1435. 10.1021/jacs.6b12581 28094931

[B48] Liu J. Xue Y. Wang Z. Xu Z.-Q. Zheng C. Weber B. (2016). Two-Dimensional CH3NH3PbI3 Perovskite: Synthesis and Optoelectronic Application. ACS Nano 10, 3536–3542. 10.1021/acsnano.5b07791 26910395

[B49] Liu M. Johnston M. B. Snaith H. J. (2013). Efficient Planar Heterojunction Perovskite Solar Cells by Vapour Deposition. Nature 501, 395–398. 10.1038/nature12509 24025775

[B50] Liu R. Zhang J. Zhou H. Song Z. Song Z. Grice C. R. (2020). Solution‐Processed High‐Quality Cesium Lead Bromine Perovskite Photodetectors with High Detectivity for Application in Visible Light Communication. Adv. Opt. Mater. 8, 1901735. 10.1002/adom.201901735

[B51] Loi H. L. Cao J. Guo X. Liu C. K. Wang N. Song J. (2020). Gradient 2D/3D Perovskite Films Prepared by Hot-Casting for Sensitive Photodetectors. Adv. Sci. (Weinh) 7 (22), 2000776. 10.1002/advs.202000776 32714769 PMC7375231

[B52] Malinkiewicz O. Yella A. Lee Y. H. Espallargas G. M. Graetzel M. Nazeeruddin M. K. (2014). Perovskite Solar Cells Employing Organic Charge-Transport Layers. Nat. Photon. 8, 128–132. 10.1038/nphoton.2013.341

[B53] Mitzi D. B. Dimitrakopoulos C. D. Kosbar L. L. (2001). Structurally Tailored Organic−Inorganic Perovskites: Optical Properties and Solution-Processed Channel Materials for Thin-Film Transistors. Chem. Mater. 13, 3728–3740. 10.1021/cm010105g

[B54] NREL Best Research-Cell Efficiencies [Online] (2020). Available at: https://www.nrel.gov/pv/assets/pdfs/cell-pv-eff-emergingpv.20200919.pdf (accessed September 25, 2020).

[B55] Pan X. Y. Zhang J. Q. Zhou H. Liu R. Wu D. Wang R. (2021). Single-Layer ZnO Hollow Hemispheres Enable High-Performance Self-Powered Perovskite Photodetector for Optical Communication. Nano-Micro Lett. 2 (13), 70. 10.1007/s40820-021-00596-5 PMC8187591 34138321

[B56] Pan Y. Y. Wang H. L. Li X. G. Zhang X. Liu F Peng M. (2020). Detection Range Extended 2D Ruddlesden–Popper Perovskite Photodetectors. J. Mater. Chem. C. 1 (20), 3359–3366. 10.1039/c9tc06109f

[B57] Quan L. N. Zhao Y. García de Arquer F. P. Sabatini R. Walters G. Voznyy O. (2017). Tailoring the Energy Landscape in Quasi-2D Halide Perovskites Enables Efficient Green-Light Emission. Nano Lett. 17, 3701–3709. 10.1021/acs.nanolett.7b00976 28475344

[B58] Seo J. Park S. Chan Kim Y. Jeon N. J. Noh J. H. Yoon S. C. (2014). Benefits of Very Thin PCBM and LiF Layers for Solution-Processed P-I-N Perovskite Solar Cells. Energy Environ. Sci. 7, 2642–2646. 10.1039/c4ee01216j

[B59] Shang Y. Li G. Liu W. Ning Z. (2018). Quasi-2D Inorganic CsPbBr3Perovskite for Efficient and Stable Light-Emitting Diodes. Adv. Funct. Mater. 28, 1801193. 10.1002/adfm.201801193

[B60] Si J. Liu Y. He Z. Du H. Du K. Chen D. (2017). Efficient and High-Color-Purity Light-Emitting Diodes Based on *In Situ* Grown Films of CsPbX3 (X = Br, I) Nanoplates with Controlled Thicknesses. ACS Nano 11, 11100–11107. 10.1021/acsnano.7b05191 29045791

[B61] Smith I. C. Hoke E. T. Solis-Ibarra D. McGehee M. D. Karunadasa H. I. (2014). A Layered Hybrid Perovskite Solar-Cell Absorber with Enhanced Moisture Stability. Angew. Chem. Int. Ed. 53, 11232–11235. 10.1002/anie.201406466 25196933

[B62] Song J. Xu L. Li J. Xue J. Dong Y. Li X. (2016). Monolayer and Few-Layer All-Inorganic Perovskites as a New Family of Two-Dimensional Semiconductors for Printable Optoelectronic Devices. Adv. Mater. 28, 4861–4869. 10.1002/adma.201600225 27110705

[B63] Song Q. Wang Y. Vogelbacher F. Zhan Y. Zhu D. Lan Y. (2021). Moiré Perovskite Photodetector toward High‐Sensitive Digital Polarization Imaging. Adv. Energ. Mater. 11, 2100742. 10.1002/aenm.202100742

[B64] Stoumpos C. C. Malliakas C. D. Kanatzidis M. G. (2013). Semiconducting Tin and Lead Iodide Perovskites with Organic Cations: Phase Transitions, High Mobilities, and Near-Infrared Photoluminescent Properties. Inorg. Chem. 52, 9019–9038. 10.1021/ic401215x 23834108

[B65] Stranks S. D. Eperon G. E. Grancini G. Menelaou C. Alcocer M. J. P. Leijtens T. (2013). Electron-Hole Diffusion Lengths Exceeding 1 Micrometer in an Organometal Trihalide Perovskite Absorber. Science 342, 341–344. 10.1126/science.1243982 24136964

[B66] Sun S. Salim T. Mathews N. Duchamp M. Boothroyd C. Xing G. (2014). The Origin of High Efficiency in Low-Temperature Solution-Processable Bilayer Organometal Halide Hybrid Solar Cells. Energ. Environ. Sci. 7, 399–407. 10.1039/c3ee43161d

[B67] Tan Z. Wu Y. Hong H. Yin J. Zhang J. Lin L. (2016). Two-Dimensional (C4H9NH3)2PbBr4 Perovskite Crystals for High-Performance Photodetector. J. Am. Chem. Soc. 138, 16612–16615. 10.1021/jacs.6b11683 27966926

[B68] Tanaka K. Takahashi T. Ban T. Kondo T. Uchida K. Miura N. (2003). Comparative Study on the Excitons in lead-halide-based Perovskite-type Crystals CH3NH3PbBr3 CH3NH3PbI3. Solid State. Commun. 127, 619–623. 10.1016/s0038-1098(03)00566-0

[B69] Tian Y. Zhou C. Worku M. Wang X. Ling Y. Gao H. (2018). Highly Efficient Spectrally Stable Red Perovskite Light‐Emitting Diodes. Adv. Mater. 30, 1707093. 10.1002/adma.201707093 29602181

[B70] Tong G. Geng X. Yu Y. Yu L. Xu J. Jiang Y. (2017). Rapid, Stable and Self-Powered Perovskite Detectors via a Fast Chemical Vapor Deposition Process. RSC Adv. 7, 18224–18230. 10.1039/c7ra01430a

[B71] Tong Y. Yao E.-P. Manzi A. Bladt E. Wang K. Döblinger M. (2018). Spontaneous Self-Assembly of Perovskite Nanocrystals into Electronically Coupled Supercrystals: Toward Filling the Green Gap. Adv. Mater. 30, 1801117. 10.1002/adma.201801117 29870579

[B72] Tsai H. Nie W. Blancon J.-C. Stoumpos C. C. Asadpour R. Harutyunyan B. (2016). High-efficiency Two-Dimensional Ruddlesden-Popper Perovskite Solar Cells. Nature 536, 312–316. 10.1038/nature18306 27383783

[B73] Tsai H. Nie W. Blancon J. C. Stoumpos C. C. Soe C. M. M. Yoo J. (2018). Stable Light‐Emitting Diodes Using Phase‐Pure Ruddlesden-Popper Layered Perovskites. Adv. Mater. 30, 1704217. 10.1002/adma.201704217 29314326

[B74] Völker S. F. Collavini S. Delgado J. L. (2015). Organic Charge Carriers for Perovskite Solar Cells. ChemSusChem 8, 3012–3028. 10.1002/cssc.201500742 26311591

[B75] Wang Jiaojiao 王. Zhao Zeping, Liu Jianguo. (2018). Research Progress and Development Trend of Balanced Photodetectors 55, 100001. 10.3788/lop55.100001

[B76] Wang N. Cheng L. Ge R. Zhang S. Miao Y. Zou W. (2016). Perovskite Light-Emitting Diodes Based on Solution-Processed Self-Organized Multiple Quantum wells. Nat. Photon 10, 699–704. 10.1038/nphoton.2016.185

[B77] Wang Q. Shao Y. Dong Q. Xiao Z. Yuan Y. Huang J. (2014). Large Fill-Factor Bilayer Iodine Perovskite Solar Cells Fabricated by a Low-Temperature Solution-Process. Energ. Environ. Sci. 7, 2359–2365. 10.1039/c4ee00233d

[B78] Wang S. Chen Y. Yao J. J. Zhao G. Li L. Zou G. (2021). Wafer-sized 2D Perovskite Single crystal Thin Films for UV Photodetectors. J. Mater. Chem. C 2 (25), 6498–6506. 10.1039/d1tc00408e

[B79] Wang Yan, Zhang Rui. (2016). Photo Detector Characteristics Effect on TDLAS Gas Detection 36, 0230002. 10.3788/aos201636.0230002

[B80] Wang Z. Lin Q. Chmiel F. P. Sakai N. Herz L. M. Snaith H. J. (2017). Efficient Ambient-Air-Stable Solar Cells with 2D-3D Heterostructured Butylammonium-Caesium-Formamidinium lead Halide Perovskites. Nat. Energ. 2, 17135. 10.1038/nenergy.2017.135

[B81] Wojciechowski K. Saliba M. Leijtens T. Abate A. Snaith H. J. (2014). Sub-150 °C Processed Meso-Superstructured Perovskite Solar Cells with Enhanced Efficiency. Energ. Environ. Sci. 7, 1142–1147. 10.1039/c3ee43707h

[B82] Wu C. Wu T. Yang Y. G. McLeod J. A. Wang Y. Zou Y. (2019). Alternative Type 2D-3D Lead Halide Perovskite with Inorganic Sodium Ions as Spacer for High Performance Light Emitting Diodes. ACS Nano 2, 1645–1654. 10.1021/acsnano.8b07632 30604954

[B83] Wu D. Zhou H. Song Z. Zheng M. Liu R. Pan X. (2020). Welding Perovskite Nanowires for Stable, Sensitive, Flexible Photodetectors. ACS Nano 14, 2777–2787. 10.1021/acsnano.9b09315 31904225

[B84] Wu T. Yang Y. Zou Y. Wang Y. Wu C. Han Y. (2018). Nanoplatelet Modulation in 2D/3D Perovskite Targeting Efficient Light-Emitting Diodes. Nanoscale 10, 19322–19329. 10.1039/c8nr04896g 30324959

[B85] Xia M. L. Yuan J. H. Luo J. J. Pan W. Wu H. Xue K. h. (2020). Two-dimensional Perovskites as Sensitive Strain Sensors. J. Mater. Chem. C 1 (27), 3814–3820. 10.1039/c9tc06437k

[B86] Xiao M. Huang F. Huang W. Dkhissi Y. Zhu Y. Etheridge J. (2014). A Fast Deposition-Crystallization Procedure for Highly Efficient Lead Iodide Perovskite Thin-Film Solar Cells. Angew. Chem. Int. Ed. 53, 9898–9903. 10.1002/anie.201405334 25047967

[B87] Xiao Z. Bi C. Shao Y. Dong Q. Wang Q. Yuan Y. (2014). Efficient, High Yield Perovskite Photovoltaic Devices Grown by Interdiffusion of Solution-Processed Precursor Stacking Layers. Energ. Environ. Sci. 7, 2619–2623. 10.1039/c4ee01138d

[B88] Xiao Z. Kerner R. A. Zhao L. Tran N. L. Lee K. M. Koh T.-W. (2017). Efficient Perovskite Light-Emitting Diodes Featuring Nanometre-Sized Crystallites. Nat. Photon 11, 108–115. 10.1038/nphoton.2016.269

[B89] Xu Z. Mitzi D. B. Dimitrakopoulos C. D. Maxcy K. R. (2003). Semiconducting Perovskites (2-XC6H4C2H4NH3)2SnI4(X = F, Cl, Br): Steric Interaction between the Organic and Inorganic Layers. Inorg. Chem. 42, 2031–2039. 10.1021/ic0261474 12639138

[B90] Yan J. Qiu W. Wu G. Heremans P. Chen H. (2018). Recent Progress in 2D/quasi-2D Layered Metal Halide Perovskites for Solar Cells. J. Mater. Chem. A. 6, 11063–11077. 10.1039/c8ta02288g

[B91] Yan Peiqin, Meng Wendong, Wang Yurong, Li Zhaohui, Tao Yuliang, Peng Huan (2017). Si-APD Single-Photon Detector with High Stability Based on Auto-Compensation of Temperature Drift 54, 080403. 10.3788/lop54.080403

[B92] Yang Hua, Cao Yang, He Junhui, Yang Qiaowen (2015). Research Progress in Graphene-Based Infrared Photodetectors 52, 110003. 10.3788/lop52.110003

[B93] Yang X. Zhang X. Deng J. Chu Z. Jiang Q. Meng J. (2018a). Efficient green Light-Emitting Diodes Based on Quasi-Two-Dimensional Composition and Phase Engineered Perovskite with Surface Passivation. Nat. Commun. 9, 570. 10.1038/s41467-018-02978-7 29422600 PMC5805756

[B94] Yang Y. You J. B. Lei M. (2018b). Efficient and Stable Perovskite Solar Cells with All Solution Processed Metal Oxide Transporting Layers. United States Patent Application Publication.

[B95] Yao K. Wang X. Xu Y.-x. Li F. (2015). A General Fabrication Procedure for Efficient and Stable Planar Perovskite Solar Cells: Morphological and Interfacial Control by In-Situ-Generated Layered Perovskite. Nano Energy 18, 165–175. 10.1016/j.nanoen.2015.10.010

[B96] Yao K. Wang X. Xu Y.-x. Li F. Zhou L. (2016). Multilayered Perovskite Materials Based on Polymeric-Ammonium Cations for Stable Large-Area Solar Cell. Chem. Mater. 28, 3131–3138. 10.1021/acs.chemmater.6b00711

[B97] Ye T. Bruno A. Han G. Koh T. M. Li J. Jamaludin N. F. (2018). Efficient and Ambient-Air-Stable Solar Cell with Highly Oriented 2D@3D Perovskites. Adv. Funct. Mater. 28, 1801654. 10.1002/adfm.201801654

[B98] Yella A. Lee H.-W. Tsao H. N. Yi C. Chandiran A. K. Nazeeruddin M. K. (2011). Porphyrin-Sensitized Solar Cells with Cobalt (II/III)-Based Redox Electrolyte Exceed 12 Percent Efficiency. Science 334, 629–634. 10.1126/science.1209688 22053043

[B99] Yuan M. Quan L. N. Comin R. Walters G. Sabatini R. Voznyy O. (2016). Perovskite Energy Funnels for Efficient Light-Emitting Diodes. Nat. Nanotech 11, 872–877. 10.1038/nnano.2016.110 27347835

[B100] Zhang F. Lu H. P. Tong J. H. Berry J. J. Beard M. C. Zhu K. (2020). Advances in Two-Dimensional Organic–Inorganic Hybrid Perovskites. Energ. Environ. Sci. 2 (18), 1154–1186. 10.1039/c9ee03757h

[B101] Zhang F. Song J. Zhang L. Niu F. Hao Y. Zeng P. (2016). Film-through Large Perovskite Grains Formation via a Combination of Sequential thermal and Solvent Treatment. J. Mater. Chem. A. 4, 8554–8561. 10.1039/c6ta03115c

[B102] Zhang H. Azimi H. Hou Y. Ameri T. Przybilla T. Spiecker E. (2014). Improved High-Efficiency Perovskite Planar Heterojunction Solar Cells via Incorporation of a Polyelectrolyte Interlayer. Chem. Mater. 26, 5190–5193. 10.1021/cm502864s

[B103] Zhang S. Yi C. Wang N. Sun Y. Zou W. Wei Y. (2017b). Efficient Red Perovskite Light-Emitting Diodes Based on Solution-Processed Multiple Quantum Wells. Adv. Mater. 29, 1606600. 10.1002/adma.201606600 28417480

[B104] Zhang X. Ren X. Liu B. Munir R. Zhu X. Yang D. (2017c). Stable High Efficiency Two-Dimensional Perovskite Solar Cells via Cesium Doping. Energ. Environ. Sci. 10, 2095–2102. 10.1039/c7ee01145h

[B105] Zhang X. Wu G. Yang S. Fu W. Zhang Z. Chen C. (2017a). Vertically Oriented 2D Layered Perovskite Solar Cells with Enhanced Efficiency and Good Stability. Small 13, 1700611. 10.1002/smll.201700611 28692766

[B106] Zhang Y. Liu Y. Xu Z. Yang Z. Liu S. F. (2020a). 2D Perovskite Single Crystals with Suppressed Ion Migration for High-Performance Planar-type Photodetectors. Small 16 (22), e2003145. 10.1002/smll.202003145 32996288

[B107] Zhao W. J. (2011). Developments in Technology of Photomultipliers. Optoelectronic Tech. 3, 145–148.

[B108] Zhou H. Chen Q. Li G. Luo S. Song T.-b. Duan H.-S. (2014). Interface Engineering of Highly Efficient Perovskite Solar Cells. Science 345, 542–546. 10.1126/science.1254050 25082698

[B109] Zhou H. Song Z. Grice C. R. Chen C. Yang X. Wang H. (2018a). Pressure-Assisted Annealing Strategy for High-Performance Self-Powered All-Inorganic Perovskite Microcrystal Photodetectors. J. Phys. Chem. Lett. 9, 4714–4719. 10.1021/acs.jpclett.8b01960 30066567

[B110] Zhou H. Song Z. Wang C. Grice C. R. Song Z. Zhao D. (2018b). Double Coating for the Enhancement of the Performance in a MA0.7FA0.3PbBr3 Photodetector. ACS Photon. 5, 2100–2105. 10.1021/acsphotonics.8b00562

[B111] Zhou N. Shen Y. Li L. Tan S. Liu N. Zheng G. (2017). Exploration of Crystallization Kinetics in Quasi Two-Dimensional Perovskite and High Performance Solar Cells. J. Am. Chem. Soc. 140, 459–465. 10.1021/jacs.7b11157 29243924

